# Unraveling the Role of Thyroid Hormones in Seasonal Neuroplasticity in European Starlings (*Sturnus vulgaris*)

**DOI:** 10.3389/fnmol.2022.897039

**Published:** 2022-06-28

**Authors:** Jasmien E. M. J. Orije, Sander R. Raymaekers, Gaurav Majumdar, Geert De Groof, Elisabeth Jonckers, Gregory F. Ball, Marleen Verhoye, Veerle M. Darras, Annemie Van der Linden

**Affiliations:** ^1^Bio-Imaging Lab, Faculty of Pharmaceutical, Biomedical and Veterinary Sciences, University of Antwerp, Antwerp, Belgium; ^2^μNEURO Research Centre of Excellence, University of Antwerp, Antwerp, Belgium; ^3^Laboratory of Comparative Endocrinology, Biology Department, KU Leuven, Leuven, Belgium; ^4^Department of Psychology, Neuroscience and Cognitive Science Program, University of Maryland, College Park, College Park, MD, United States

**Keywords:** thyroid hormone, testosterone, MRI, DTI, neuroplasticity, songbird, European starling (*Sturnus vulgaris*)

## Abstract

Thyroid hormones clearly play a role in the seasonal regulation of reproduction, but any role they might play in song behavior and the associated seasonal neuroplasticity in songbirds remains to be elucidated. To pursue this question, we first established seasonal patterns in the expression of thyroid hormone regulating genes in male European starlings employing *in situ* hybridization methods. Thyroid hormone transporter LAT1 expression in the song nucleus HVC was elevated during the photosensitive phase, pointing toward an active role of thyroid hormones during this window of possible neuroplasticity. In contrast, DIO3 expression was high in HVC during the photostimulated phase, limiting the possible effect of thyroid hormones to maintain song stability during the breeding season. Next, we studied the effect of hypothyroidism on song behavior and neuroplasticity using *in vivo* MRI. Both under natural conditions as with methimazole treatment, circulating thyroid hormone levels decreased during the photosensitive period, which coincided with the onset of neuroplasticity. This inverse relationship between thyroid hormones and neuroplasticity was further demonstrated by the negative correlation between plasma T3 and the microstructural changes in several song control nuclei and cerebellum. Furthermore, maintaining hypothyroidism during the photostimulated period inhibited the increase in testosterone, confirming the role of thyroid hormones in activating the hypothalamic–pituitary–gonadal (HPG) axis. The lack of high testosterone levels influenced the song behavior of hypothyroid starlings, while the lack of high plasma T4 during photostimulation affected the myelination of several tracts. Potentially, a global reduction of circulating thyroid hormones during the photosensitive period is necessary to lift the brake on neuroplasticity imposed by the photorefractory period, whereas local fine-tuning of thyroid hormone concentrations through LAT1 could activate underlying neuroplasticity mechanisms. Whereas, an increase in circulating T4 during the photostimulated period potentially influences the myelination of several white matter tracts, which stabilizes the neuroplastic changes. Given the complexity of thyroid hormone effects, this study is a steppingstone to disentangle the influence of thyroid hormones on seasonal neuroplasticity.

## Introduction

Seasonal changes in steroid hormone concentrations such as testosterone play a central role in the regulation of song behavior and the associated neuroplasticity in seasonal songbirds (Balthazart et al., 2010). For example, testosterone acting in the preoptic area can increase the motivation to sing, resulting in activity-induced neuroplasticity, including increased forebrain song control nuclei volume (Alward et al., 2013) and stronger connections between the song control nuclei (Orije et al., 2020). In several songbird species, testosterone is known to be necessary for the crystallization of song (Marler et al., 1988; Korsia and Bottjer, 1991; Williams et al., 2003), while castration increases syllable’s entropy and decreases the song similarity and stereotypy (Wang et al., 2014). Male canaries sing a stable song during the breeding season, when testosterone levels are high. As canaries become photorefractory in fall, their testosterone levels decrease and their song becomes plastic again (Nottebohm et al., 1987; Voigt and Leitner, 2008; Alward et al., 2014). These studies demonstrate that testosterone plays a key role in stimulating songbirds of various species to sing at higher rates and to crystalize or stabilize their song, and that these behavioral changes are associated with structural changes in their song control system. The presence of aromatase (the enzyme responsible for the local production of estrogens through the aromatization of androgens), androgen and estrogen receptors in several song control nuclei is consistent with the notion that testosterone can act directly in these key song control nuclei to induce changes in morphology and physiology. However, several studies have shown that even in the absence or presence of very low concentrations of gonadal testosterone seasonal neuroplasticity can still occur, indicating that there are other factors that regulate or influence seasonal neuroplasticity (Smith et al., 1997; Bentley et al., 1999; Dawson et al., 2001). One of the candidate factors that might regulate seasonal plasticity in brain and behavior are thyroid hormones. The avian thyroid gland produces thyroxine (T4) as well as a small amount of triiodothyronine (T3), which are both released into the bloodstream. Thyroid hormone activity is subsequently regulated at multiple levels. Several thyroid hormone transporters, including monocarboxylate transporters (MCT8 and MCT10), Na-independent organic anion transporting polypeptide 1C1 (OATP1C1), and L-type amino acid transporter 1 (LAT1) facilitate transport of T3 and T4 across the cell membrane (Heuer and Visser, 2009). Once in the cell, the activity of thyroid hormones can be locally adjusted by deiodinases. Deiodinase 2 (DIO2) converts the prohormone T4 into its more bioactive form T3 and creates the main source of T3 within the brain. On the contrary, deiodinase 3 (DIO3) inactivates T4 and T3 to reverse T3 (rT3) and T2, respectively (Gereben et al., 2008). Thyroid hormones mostly operate by modulating gene expression as a result of binding of T3 to nuclear thyroid hormone receptors alpha or beta (THRA or THRB). These receptors associate with thyroid hormone response elements in the promotor region and induce or repress gene expression, depending on the gene (Gil-Ibanez et al., 2017).

Thyroid hormone levels change seasonally and are known to play a role in reproductive maturation and maintaining the photorefractory state in seasonally breeding birds (Bentley et al., 1997, 2000). They closely interact with the hypothalamus-pituitary-gonadal (HPG) axis. One of the first things to change after exposure to a single long day of Japanese quail is the local upregulation of thyroid stimulating hormone (TSH) and DIO2 and downregulation of DIO3 in the pars tuberalis of the pituitary gland. This results in a local increase in T3 in the mediobasal hypothalamus, which causes gonadotropin releasing hormone (GnRH) release from morphologically changed GnRH nerve terminals and activates the rest of the HPG-axis (Yoshimura et al., 2003; Watanabe et al., 2007; Nakao et al., 2008). Furthermore, thyroid hormones are known to play an important role during neuronal development and are suggested to influence critical period learning (Bernal, 2000; Yamaguchi et al., 2012; Batista and Hensch, 2019). However, in starlings under natural daylight conditions, the photoperiodic mechanism for thyroid hormones to regulate gonadal steroid hormone production was not confirmed, showing that our understanding of thyroid hormones, reproduction and seasonality is still inadequate (Bentley et al., 2013). To date the exact role of thyroid hormones in seasonal neuroplasticity in song and the song system has not been investigated yet.

We hypothesize that thyroid hormones contribute to the seasonal neuroplasticity related to song behavior. If thyroid hormones are indeed able to actively influence seasonal neuroplasticity, we expect in the first place expression of thyroid hormone receptors, transporters and deiodinases in the song bird brain and more specifically in the song control system. The first report describing a role of thyroid hormone regulating genes during development of the song control system and song learning in the zebra finch brain came from our coauthors (Raymaekers et al., 2017). They showed that DIO2 and LAT1 expression in several song control nuclei remained high during the sensory and sensorimotor phase of song learning in male zebra finches, suggesting that thyroid hormones are key players in this process. In line with the role of thyroid hormones in song development in zebra finches, we expected that the expression of thyroid hormone regulating genes could change seasonally in starlings and have differential effects in regulating song and neuroplasticity across the seasons. Once we had established the spatial and temporal expression patterns of thyroid hormone regulating genes (and thyroid hormone plasma levels) during different photoperiods, we induced hypothyroidism during the photosensitive and photostimulated stage to investigate the impact on song production and seasonal neuroplasticity using *in vivo* MRI. In a previous MRI study, we showed that seasonally increased neuroplasticity starts during the photosensitive period, which acts as a sensitive window for multisensory neuroplasticity as it shows involvement of the song control, visual and auditory system and even the cerebellum (Orije et al., 2021a). Furthermore, *in vivo* MRI is a powerful tool to study neural substrate changes associated with singing behavior or hormonal changes (Hamaide et al., 2020; Orije et al., 2020, 2021a).

In short, this study aimed to answer the following questions: (1) Are thyroid hormone regulatory genes expressed in the song control system of male starlings? (2) Does their expression change seasonally? (3) How does thyroid hormone deprivation affect seasonal neuroplasticity and song behavior in male starlings during the photosensitive and photostimulated phase? (4) Does thyroid hormone manipulation affect the HPG-axis just like in Japanese quail?

## Materials and Methods

### Subjects and Experimental Setup

**FIGURE 1:**
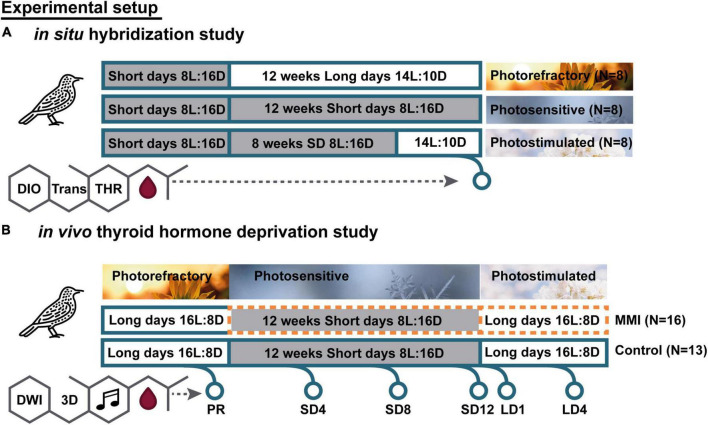
Overview experimental setup of *in situ* hybridization **(A)** and *in vivo* thyroid hormone deprivation **(B)** study. For *in situ* hybridization, all birds were photosensitive after being on a short day photoperiod. These birds were divided into three groups with different photoperiodic manipulation: 12 weeks of long days to induce the photorefractory state (PR), 12 weeks of short days to maintain photosensitive state, 8 weeks of short days followed by 4 weeks of long days to induce photostimulated state. For each photoperiod we analyzed different thyroid hormone regulators: deiodinases (DIO), thyroid hormone transporters (Trans), and thyroid hormone receptors (THR). For the *in vivo* manipulation study, we monitored individual starlings as they went through different photoperiods sequentially and diffusion weighted images (DWI) and 3D MRI scans were acquired at the indicated time points. Short days (SD) are indicated by a gray box. Methimazole (MMI) treatment is indicated by an orange dotted line.

Wild European starlings (*Sturnus vulgaris*) used in the *in situ* hybridization (ISH) experiment were caught in Baltimore, United States during mid-late winter 2013 and subsequent procedures were performed by the research group of prof. Gregory Ball. Twenty-four male adult starlings were divided in three groups of eight, and were housed under different photoperiodic regimes in order to induce photosensitive, photostimulated and photorefractory states (Figure 1A). All birds were initially maintained on short day lengths (8 h of light, 16 h of dark, 8L:16D) to keep them in a photosensitive state. Photoperiod manipulations were performed over a period of 12 weeks. Group 1 was immediately put on long day lengths (14L:10D) for 12 weeks so they would be photorefractory by the end of photoperiod manipulation. Group 2 was kept on 8L:16D for 12 weeks so that they would remain photosensitive. Group 3 was kept on 8L:16D for 8 weeks, then switched to long day lengths (14L:10D) for 4 weeks to make them photostimulated. In order to control for handling stress associated with transferring group 2 to a different photoperiod, all groups were transferred into a new cage located within the same room. Birds remained with the same cage mates throughout the entire experiment. No females were present in any group. While studies showed that the presence of females influences neurological attributes and singing rate in canaries (Alward et al., 2014; Shevchouk et al., 2017), and even slightly increases the growth of the sparrow HVC (used as a proper name) and the robust nucleus of arcopallium (RA) compared to isolated birds, photoperiod is still the main instigator of song control nucleus growth (Tramontin et al., 1999).

Food and water was supplied *ad libitum*. In order to verify sex, exploratory laparotomies were performed prior to placing birds into their experimental groups. Under isoflurane anesthesia, a small incision was made on the bird’s left side and gonads were visually ID’d. The incision was closed with nylon sutures, and birds were allowed to recover for 1 week. After 1 week, they were placed into the appropriate experimental groups. At the end of the 12 week period, all birds were anesthetized *via* injection of secobarbital followed by cardiac perfusion with 4% paraformaldehyde. Brains were dissected out and placed into 4% paraformaldehyde. All procedures were in accordance with the animal welfare regulations of the John Hopkins University of Baltimore, MD, United States. Brains were quickly frozen, stored at –80°C and sent to the research group of Comparative Endocrinology at the KU Leuven together with blood samples, where all subsequent procedures took place.

The second experiment was performed on thirty male starlings (*Sturnus vulgaris*) that were wild caught as adults in Normandy (France) in November 2014. All animals were housed in two large indoor aviaries (L × W × H: 2.2 × 1.4 × 2.1 m) without nest boxes at the University of Antwerp. Food and water were provided *ad libitum*. Starting from January 2013, using artificial light-dark cycles, all birds were kept in a long day photoperiod (16L/8D) in order to remain photorefractory. The housing and experimental procedures were performed in agreement with the Belgian and Flemish laws and were approved by the Committee on Animal Care and Use of the University of Antwerp, Belgium (2014-52).

Starlings were divided into two groups: a hypothyroid group (*N* = 16) and a control group (*N* = 14) (Figure 1B). The study was started when all birds were photorefractory. Then they were switched from long to short (8L:16D) days to induce the return to photosensitivity. Methimazole (MMI) treatment was started in one group to induce hypothyroidism. By supplementing the drinking water with 0.05% MMI, the endogenous stock of THs gradually decreased until it was fully depleted after 2–3 weeks. MMI treatment was continued for the remainder of the experiment. After 12 weeks of short days, the photosensitive starlings were switched back to long days (16L:8D) so that they became photostimulated. In parallel, the control group was exposed to the same photoperiodic regime without receiving any hormone manipulation.

We monitored the neuroplasticity using MRI repeatedly at six different time points. The first time point was at the end of the photorefractory state (PR). After switching to short days we measured every 4 weeks to follow up the song control system plasticity during the photosensitive period (SD4, SD8, and SD12). Additionally, we measured after 1 week of long days, as it is known that exposure to 1 long day can already affect TH. Finally, we measured after 4 weeks on long days (LD4), when starlings were fully photostimulated. At each time point, songs were recorded, blood samples were taken, and MRI (DTI and 3D) was acquired. In addition, body weight and beak color were registered (Figure 1B).

### *In situ* Hybridization

30 μm thick coronal brain sections were made using a cryostat and kept free floating in cryobuffer (1.59 g/l NaH_2_PO_4_, 5.47 g/l Na_2_HPO_4_, 300 g/l sucrose, 10 g/l polyvinylpyrrolidone and 300 ml/l ethylene glycol in RNase free H_2_O) in 24 series so that each subsequent section in a series was 720 μm apart from the previous one. Sections were stored in cryobuffer at –20°C until used.

### DNA Cloning and Probe Synthesis

RNA was isolated from an adult male starling brain using TRIzol (Invitrogen) and converted to cDNA using Superscript III reverse transcriptase (Invitrogen). Based on the available starling or homologous zebra finch or chicken sequences on the NCBI website, primers were designed to amplify 800–1,000 base pair segments of the different TH regulators. The primers used for each gene are summarized in Table 1. The DNA sequences were amplified using PCR (10 min 95°C followed by 35 cycles of 15 s 95°C, 15 s 60°C, 2 min 72°C). Amplified sequences were cloned into the pGEM-T easy vector (Promega) using the associated kit. The produced plasmids were used for transformation of competent JM109 *E. coli* cells (Promega). Cells were cultured overnight and the plasmid was purified using a Plasmid Miniprep kit (Invitrogen). Plasmid sequences were sequenced by Sanger-sequencing to determine insert orientation and one batch per orientation was retained to provide template for both sense and antisense probe. Cloning was successful except for the sequence of OATP1C1. Therefore, we instead used the corresponding probe for zebra finch (Raymaekers et al., 2017) which has 94% sequence homology with the corresponding starling sequence, which is sufficient to generate specific signal. Plasmids were cut using *Spe*I endonuclease (Invitrogen) and purified using ethanol precipitation. Linearized plasmid was then used as template to produce digoxigenin (DIG-) labeled probes using T7 RNA polymerase (Invitrogen) and a DIG labeling mix (Invitrogen). The resulting probes were again purified using ethanol precipitation first followed by a Sephadex G-25 column (Roche). Probes were evaluated using a dot blot and were held at 20°C in 50% formamide until use.

**TABLE 1:** Primers for cloning of partial sequences of thyroid hormone regulators in starling.

Gene	Forward primer (5′–3′)	Reverse primer (5′–3′)	NCBI accession no.
*DIO2*	ATAGCCAAGGGCAGTGATGG	CACCCTACCTGCTTGACAGC	XM_014869830
*DIO3*	GTGTCCGACTCCAACTGCAT	TGGTCTAGCCAGCTCCTCAG	XM_014871958
*MCT8*	ATCGTCAGCATCTTCACCGA	ATGGTGGTGAAGAAGCCATC	XM_014877151
*MCT10*	AGACCTGTTTGGCTGTCGAA	GACAGCAACGAGAGCTCCAA	XM_014879264
*OATP1C1*	CAAACAGCACAGTCTTGCCC	CAGCTATCAGGTAGCCCAGC	XM_002196271.2
*LAT1*	CGCCTACATGCTGGAGGTTT	AGAACAGCCGGGAAGAAGTG	XM_014874486
*THRA*	AGATGAACAGTGTGTGGTGT	ACTTGCCGAGGTCGAAGATG	XM_014885513
*THRB*	TGCCAGGAATGTCGCTTCAA	AGTGTTCGAATGCCAGGAGG	XM_014887263

### *In situ* Hybridization Staining

The ISH protocol used for detection of thyroid hormone regulator genes in starling closely resembled the protocol used for zebra finch (Raymaekers et al., 2017). From one series of telencephalon sections, those containing song control nuclei were selected and placed individually in wells of a 24-well plate. This was on average three sections for Area X, two for the lateral magnocellular nucleus of the anterior nidopallium (LMAN), two for HVC and two for RA. Of each photoperiod, one brain was omitted from staining due to cutting or freezing damage; thus the number of birds (N) stained for each photoperiod was 7. ISH was performed with both anti-sense and sense (negative control) probes. Sections were rinsed in PBS and acetylated (13.5 ml/l triethanolamine and 2.5 ml/l acetic anhydride in RNase free H_2_O) for 10 min. Sections were then rinsed in SSPE buffer (174 g/l NaCl, 24 g/l NaH_2_PO_4_, 5.84 g/l EDTA in H_2_O) and incubated overnight at 65°C in hybridization buffer (50% formamide, 2 g/l yeast tRNA, 1 g/l bovine serum albumin, 1 g/l polyadenylic acid in SSPE buffer) with 100 ng DIG-labeled probe targeting the gene of interest. The next day excess probe was washed off by rinsing in 50% formamide in SSPE for 70 min and then twice in 0.05X SSPE buffer for 30 min. Sections were then rinsed in TN buffer (12.12 g/l Tris-HCl, 8.766 g/l NaCl, pH 7.5) with 0.3% Triton-X 100, blocked for 30 min with blocking buffer (5 g/l casein and 1% skimmed milk) and incubated for 3 h with anti-DIG-alkaline phosphatase antibody (Roche) diluted 1/600 in blocking buffer. Excess antibody was washed off by rinsing twice in alkaline TN buffer (12.12 g/l Tris- HCl, 8.766 g/l NaCl, pH 9.5) and the antibody was visualized by incubation in 4.5% nitro-blue tetrazolium (Promega) and 3.5% 5-bromo-4-chloro-30-indolyphosphate (Promega) in alkaline TN. Sections were kept in NBT/BCIP buffer until sufficient staining was attained, were then rinsed in TN buffer and mounted on glass slides (Thermo Fisher Scientific) and dried. Finally, slides were coverslipped with Mowiol and stored in the dark at room temperature.

### Nissl Staining

Of three birds of each photoperiod, 2 HVC sections, 720 μm apart, were stained with cresyl violet. Sections were rinsed in PBS, incubated in 1% cresyl violet in H_2_O for 1 min and then rinsed three times in H_2_O containing 2.5% acetic acid for 1 min. Sections were again rinsed in H_2_O, mounted onto glass slides, incubated in xylene for 30 s and coverslipped with DPX (Sigma).

### *In situ* Hybridization Imaging and Data Analysis

Photomicrographs in the regions of interest were taken for all markers using a stereomicroscope (Carl Zeiss). Data in HVC from the *DIO3* and *LAT1* ISH and the Nissl staining were quantified (on average two sections per bird for each marker) using Fiji software (ImageJ, National Institute of Health) and statistically analyzed using GraphPad Prism 5 (GraphPad Software, Inc.). We only analyzed one side of each section, selecting a 100,000 μm^2^ square box was selected in the middle of HVC (central on the mediolateral and dorsoventral axes through the nucleus). Inside that box, *DIO3*+ cells, *LAT1*+ cells and cells stained by cresyl violet were counted. Cells were counted as a “stained cell” if the dark signal was delineated in the form of at least a semicircle (180 degrees or more) around the center. Cell counts of both sections from one bird were then averaged to obtain 1 numerical value per bird. Signal throughout HVC was generally homogeneously spread, but for rare sections with heterogeneous signal, larger areas were analyzed and data normalized. We analyzed the differences in *DIO3*+, *LAT1*+, or Nissl stained cells between photoperiods using a linear mixed model. When appropriate, further comparisons were performed with the Bonferroni *post-hoc* test or Dunn’s *post-hoc* test respectively to analyze differences between two photoperiods.

To further evaluate the validity of the cell count method used for the *DIO3* and *LAT1* ISH, 10 random sections of both ISHs were analyzed using the semi-quantitative manner of ‘stained surface fraction’ as used for the zebra finches (Raymaekers et al., 2017). For each section, a 100,000 μm^2^ square box was selected in the middle of HVC. The image was converted to grayscale (each pixel is assigned a brightness value between 0 and 255) and a brightness threshold was manually set for each image so that dark staining was counted as “stained” while background was not. This way, the stained surface area was calculated and divided by the total area of the 100,000 μm^2^ box. The stained surface fraction values were plotted against their corresponding original cell count value. Linear regression analysis was performed and the coefficient of determination (r^2^) was calculated using Prism 5 to check for correlation.

### Blood Sample Analysis and Peripheral Physiology

At each time point of the hypothyroidism experiment blood samples were taken to assay plasma levels of T3, T4 and testosterone. In order to limit variation of plasma levels due to the time of the day, blood samples were taken at each stage within a 2 h window at noon between 11u30 and 13u30. Animals were already subdivided in small cages for individual song recordings. This allowed fast capture and blood sampling (within 5 min of capture) to minimize increase in testosterone as a result of acute stress (Van Hout et al., 2010). Birds were weighed before blood collection. The alar wing vein was punctured with a 25-gauge needle to collect 300–500 μl of blood into heparin-coated capillary tubes. After the blood collection the birds returned to their individual cages. Blood samples were centrifuged for 10 min at 2,060 g while cooling to 4°C. After centrifugation, the plasma was collected and frozen at –20°C, where the samples remained until analysis. Plasma testosterone concentrations were quantified by radioimmunoassay (RIA) using a commercial double antibody system from MP Biomedicals (Ohio). This system does not significantly cross-react with other androgens beside testosterone (5a-dihydrotestosterone: 3.4%; 5a-androstane-3b, 17b-diol: 2.2%; 11-oxo-testosterone: 2%; all other steroids: <1%) and the intra-assay coefficient of variation is 4.6–9.1%. Plasma T3 and T4 concentrations were measured by a home-assembled RIA using antibodies and standards from Byk-Sangtec Diagnostica (Germany) and tracers from PerkinElmer (Belgium). The T3 RIA had a detection limit of 2 fmol and an intra-assay coefficient of variation of 2.2%. The T4 RIA had a detection limit of 5 fmol and an intra-assay coefficient of variation of 2.8%. For the T3 RIA, cross-reactivity with T4 was 0.1–0.5%, whereas for the T4 RIA, cross-reactivity with T3 was 3.5%.

Next to the quantitative analysis of hormone plasma levels, beak color was assessed at each time point as a physiologic indicator of circulating testosterone concentration. Beak color changes from black to yellow in starlings if testosterone is present in the circulation and is a sensitive indicator for even low doses of testosterone below the detection limit of a RIA assay (Ball and Wingfield, 1987; Wingfield and Silverin, 2002). Beak color was scored from 0 to 5, where 0 represents a black beak and 5 a complete yellow beak. Scores of 1 to 4 were given to indicate the part of the beak that was colored yellow, ranging from 20% (score 1), 40% (score 2), 60% (score 3) up to 80% (score 4) starting from the base of the beak.

### Song Analysis

At each photoperiod songs were recorded for 48 h using Behringer C-2 condensator microphones, connected to a PR8E multi-channel preamplifier (SM Pro audio) and “Delta Series” Breakout box (M-audio). The microphones were placed on top of individual cages. Due to technical issues, songs of birds were lost at PR and SD12 in both groups and at SD4 in the MMI treated group. Since fully isolated starlings do not sing as they would do in group context, the individual cages were placed next to the aviary. After the 48 h of song recording the starlings returned to the large aviary. Song recordings of 4 consecutive hours in the morning were analyzed using SoundExplorer (Developed by RF Jansen, University of Amsterdam). Based on the intensity and the spectral definition of the song bouts, individual song could be differentiated from background noise of the aviary. In line with prior song processing (Eens, 1997; Van Hout et al., 2012) we defined a song bout as a period of at least 5 s of song with pauses no longer than 1.5 s. Starling song consists out of 4 distinct phrases, which are mostly performed in a fixed order. The song often starts with one or several whistles (whistle phrase), followed by a section of complex phrases (variable phrases) and rapid series of clicks or rattles (rattle or click phrase), before ending the song with high frequency song elements (high frequency phrases). Song bouts containing at least three different phrases were labeled as “complete song bouts.” Analysis of the song bout length was only performed on complete songs. If there were no complete song bouts at a certain time point, the average song bout length of incomplete songs was calculated and taken as a measure of the evolution in song production.

The number of complete song bouts sung per hour within the 4-h period was taken as a measure of the song rate. We used the first 20 complete song bouts sung within this 4-h period to calculate the song bout length. This way, we avoided the overrepresentation of songs during time points where the song rate was high.

### MRI Data Acquisition

The birds were initially anesthetized using 2% Isoflurane (Isoflo^®^, Abbot Laboratories Ltd.) in a mixture of 30% O_2_ and 70% N_2_ at a flow rate of 600 ml/min. Throughout the entire imaging procedure, respiration rate was monitored with a small pneumatic sensor (SA Instruments, NY, United States) positioned under the bird. Depending on the breathing rate, the anesthetic dose was lowered, ranging between 1 and 2% isoflurane. Body temperature was monitored with a cloacal temperature probe and kept within narrow physiological ranges (41.0 ± 0.2°C) using a warm air system with a feedback unit (SA Instruments, NY, United States).

All MRI measurements were performed on a 7 T horizontal MR system (Pharmascan 70/16 US, Bruker Biospin, Germany). Each imaging session started with a T2-weighted 3D anatomical rapid acquisition with relaxation enhancement (RARE) scan [TR: 2,000 ms; TE: 11 ms; RARE factor: 8; zero-filled to a matrix of (256 × 92 × 64) with voxel resolution (0.089 × 0.25 × 0.25) mm^3^]. Subsequently, a 4 shot spin echo echo-planar imaging (SE-EPI) DTI scan [TR: 7,000 ms; TE: 23 ms; d 4 ms, D 12 ms; b-value 670 s/mm^2^; 60 diffusion gradient directions; spatial resolution: (0.179 × 0.179 × 0.35) mm^3^; 28 coronal slices] was acquired. After the imaging procedure, birds were left to recover in a warmed recovery box before returning to the aviary.

### MRI Data Processing

Diffusion data were analyzed with MRtrix3 version 3.0 (Tournier et al., 2012) following the same processing steps as described in Orije et al. (2021b). Preprocessing of the individual DW-images included the following steps: denoising (Veraart et al., 2016), correction for Gibbs ringing (Kellner et al., 2016), motion and distortion correction using FSL (Jenkinson et al., 2012), bias field correction using ANTS (Advanced Normalization Tool; Avants et al., 2010), whole brain extraction and upsampling to isotropic voxels of 1.75 mm. These preprocessed diffusion weighted images were used to calculate individual diffusion maps [fractional anisotropy (FA), mean, axial, and radial diffusivity] and fiber orientation distribution (FOD) images. The neuroanatomical contrast of the individual FA maps allowed the delineation of different ROIs (Area X and RA) to determine their volume changes using AMIRA software (De Groof et al., 2006). The calculation of FOD images requires global intensity normalization, fiber response function estimation using the unsupervised Dhollander algorithm (Dhollander et al., 2019) and spherical deconvolution (Jeurissen et al., 2014). These FOD images were normalized to create an unbiased study-based FOD template, which involves linear and non-linear registration (Raffelt et al., 2011). Fiber density (FD) and fiber-bundle cross-section (FC) were estimated from the normalized FOD images. The transformation parameters derived from building the FOD template were also applied to the diffusion maps to warp them into the template space to perform voxel-based analysis. Next, these images were smoothed to double voxel size (3.5 × 3.5 × 3.5 mm^3^). Finally, all normalized diffusion maps were averaged to create a FA template that is used as background to display the statistical results.

### Statistical Analysis

Voxel-based statistical analyses were performed using SPM 12.^1^ Following the recommendations of McFarquhar (2019) the effect of time and the interaction between treatment group and time were assessed using a flexible factorial design in SPM on a whole brain level. Voxel-based multiple regression analysis was used to determine the correlation of changes in FA to song parameters and hormone concentrations. This analysis involved the use of a mask for song control nuclei, auditory system and their tracts, to ensure that we do not miss significant changes in relevant regions that might be obscured by the stringent correction for multiple comparisons in whole brain analysis. The significant voxel-based clusters are further explored by extracting the average diffusion and FOD-derived values (including FA, mean, axial and radial diffusivity, FD and log FC) from these statistical region of interest (ROI) clusters. Linear mixed models with *Post-hoc* Tukey’s HSD multiple comparison (*p* < 0.05) correction were performed on the ROI data using JMP Pro 15 (SAS Institute Inc., Cary, NC, 1989-2007) to assess the main effect of time, treatment group and their interaction. Since we have repeated measures, the assumption of independence used in traditional correlation techniques like Pearson correlation is violated when performing a multiple regression on longitudinal data. Traditionally this is solved by using data from only one time point. However, a lot of information on temporal changes is lost this way. Therefore, we use repeated measures correlation instead of traditional correlation techniques (Bakdash and Marusich, 2017). This way, we can assess the within-subject correlation, which gives a meaningful interpretation of how thyroid hormones, song and diffusion parameters correlate to each other on a within-subject level.

Statistical analysis of testosterone levels and song behavior was performed using linear mixed model *via* JMP Pro 15. *Post-hoc* tests were Tukey corrected for multiple comparison. Differences were considered significant for *p* < 0.05.

## Results

### Thyroid Hormone Regulators in the Song Control System: *In situ* Hybridization Results

To understand fully the role and regulation of thyroid hormone action in seasonal neuroplasticity in male starlings, we performed ISH for mRNA of all known major thyroid hormone regulators. Of these thyroid hormone regulators *DIO2*, *MCT8*, *MCT10*, and *OATP1C1* showed no visible expression in any of the photoperiods in any of the song nuclei nor in the rest of the telencephalon (data not shown).

**FIGURE 2:**
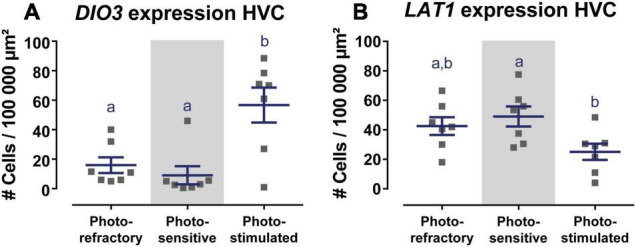
Overview of *DIO3* **(A)** and *LAT1* **(B)** expression in the HVC in the photorefractory, photosensitive and photostimulated phase. Dots represent the average number of *DIO3*+ and *LAT1*+ cells per individual bird. Horizontal bars represent the average with standard error of the mean error bars (*n* = 7). The gray area indicates the photosensitive phase. Different letters denote significant differences by comparison with each other in *post-hoc t*-tests with *p* < 0.05 (multiple comparison correction with Dunn’s *post-hoc* tests for DIO3 and Bonferroni correction for LAT1). If two time points share a common letter, *DIO3* or *LAT1* expression are not significantly different from each other.

**FIGURE 3:**
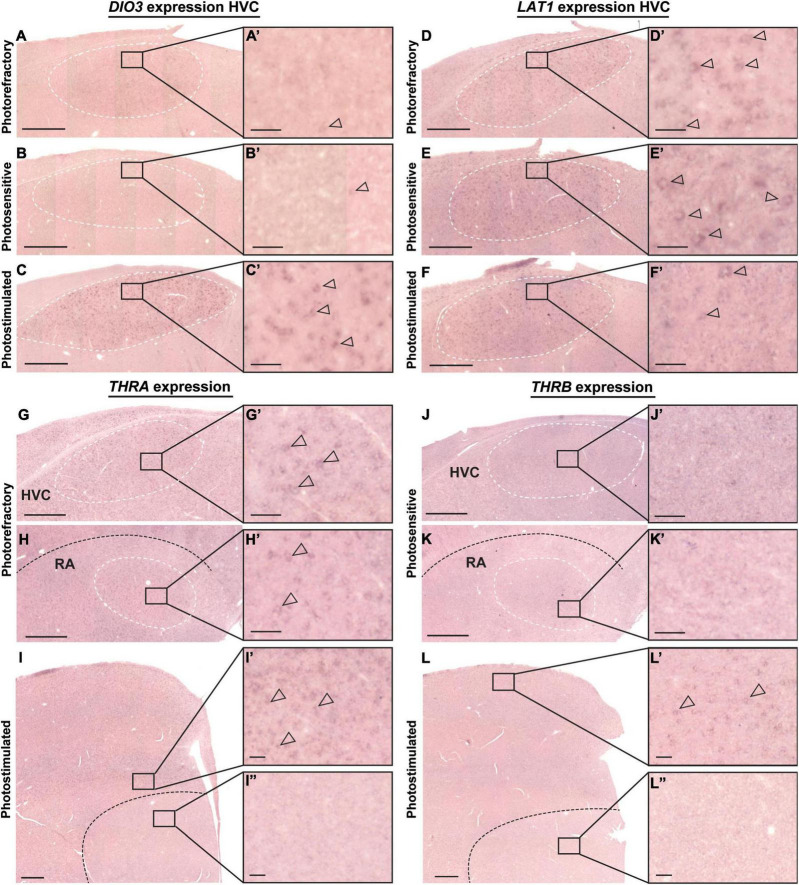
Representative images of *DIO3* **(A–C)**, *LAT1* **(D–F)**, *THRA* **(G–I)**, *THRB* **(J–L)** mRNA expression in the starling brain. *DIO3* and *LAT1* mRNA expression changes seasonally over the photorefractory **(A,D)**, photosensitive **(B,E)**, and photostimulated phase **(C,F)**. *THRA* and *THRB* mRNA expression remain unchanged during the different seasons in caudal telencephalon **(G,H,J,K)** at the level of HVC **(G,J)** and RA **(H,K)** and in the rostral telencephalon **(I,L)** at the level of LMAN **(I’)**, pallium **(L’)**, and Area X **(I”, L”)**. The inserts indicate the location of the close-ups **(A’–L’)**. The borders of HVC and RA are designated by white dashed lines. Black dashed line indicates the border between nidopallium and arcopallium in panels **(H,K)** and the border between striatum and pallium in panels **(I,L)**. Empty arrowheads designate examples of *DIO3*+, *LAT1*+, and *THRA*+ cells respectively. Scale bar in overview and inset pictures are 500 and 50 μm, respectively.

Of the main thyroid hormone activating and inactivating enzymes DIO2 and DIO3, only *DIO3* expression was detected in the HVC and varied over the different photoperiods [*F*(2, 18) = 9.69, *p* = 0.0014). Photorefractory and photosensitive birds showed little to no *DIO3*+ cells, whereas *DIO3* expression was much higher after 4 weeks of photostimulation (Figure 2A and Figures 3A–C).

In contrast to the other thyroid hormone transporters, *LAT1* showed a strong expression in HVC in all three photoperiods [*F*(2, 18) = 4.10, *p* = 0.0342] (Figures 3D–F). *Post-hoc* Bonferroni test showed that the *LAT1* expression in the HVC in the photosensitive state was significantly higher than in the photostimulated phase (Figure 2B). Furthermore, there was a low, widespread expression of LAT1 in the upper layers of the pallium both rostrally and caudally and a low expression in the arcopallium including RA. No expression was observed in other song control nuclei.

Finally, we examined the expression pattern of thyroid hormone receptors. *THRA* showed a clear and widespread expression throughout the entire pallium including HVC, RA, and LMAN, whereas no expression was detected in the striatum, including Area X (representative image shown in Figures 3G–I). In general, there was no variation in *THRA* expression over the different photoperiods. Furthermore, the *THRA* expression was not specific for the song control nuclei, since similar density and intensity of *THRA* expression was found in the surroundings of HVC, RA, and LMAN. *THRB* expression was much lower compared to *THRA* and was only found in upper layers of the pallium and caudal arcopallium but not detected in the RA or any other song control nucleus (Figures 3J–L).

The widespread expression of *THRA* makes it possible for seasonal changes in T4 and T3 to affect several song control nuclei, except striatal regions like Area X. Active seasonal changes in thyroid hormone regulating genes (*DIO3* and *LAT1*) are confined to the HVC, as the central regulator of other song control nuclei it is connected to. This suggests that the intracellular concentration of thyroid hormones is actively controlled at the level of HVC, and could play a role in seasonal song behavior.

### Validation of *in situ* Hybridization Cell Counting

To evaluate the validity of the cell count method used for the *DIO3* and *LAT1* ISH, 10 random sections of both ISHs were analyzed using the semi-quantitative manner of “stained surface fraction.” The correlation between both methods measuring expression (stained surface fraction values and original count value) r^2^ was 0.944 for the *DIO3* ISH and 0.924 for the *LAT1* ISH.

Furthermore, we investigated whether a change in cell density in the HVC across seasons could result in changes in *DIO3*+ and *LAT1*+ cell counts, by performing a Nissl staining on sections from each photoperiodic treatment in 3 birds. Cells stained by cresyl violet were counted in HVC, but numbers showed no variation over time [*F*(2, 20) = 0.44, *p* = 0.649] (Supplementary Figure 1).

### Thyroid Hormone Decrease Coincides With Onset of Seasonal Neuroplasticity

**FIGURE 4:**
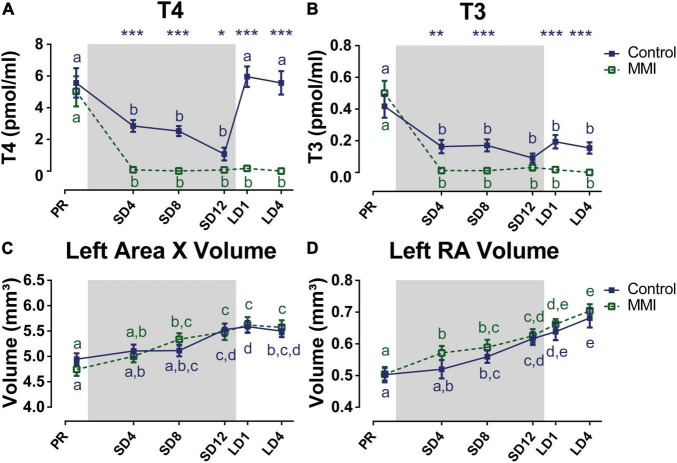
Overview of the seasonal changes in plasma levels of thyroid hormones T4 **(A)**, T3 **(B)**, and Area X **(C)**, and RA volume **(D)** in control and MMI-treated starlings. Solid and dashed lines represent the group average of control and MMI-treated starlings respectively with standard errors of the mean error bars. The gray area indicates the photosensitive period of short days (8L:16D). Different letters denote significant differences by comparison with each other in *post-hoc t*-tests with *p* < 0.05 (Tukey’s HSD correction for multiple comparisons) comparing the different time points to each other. If two time points share a common letter, the thyroid hormone levels, Area X or RA volumes are not significantly different from each other.

In a second experiment we depleted circulating thyroid hormone levels by MMI treatment to establish how hypothyroidism affects seasonal neuroplasticity during the photosensitive and photostimulated phase. At the baseline photorefractory time point there was no difference in circulating thyroid hormone concentrations between groups. MMI treatment was successful in reducing both T3 and T4 to a minimum at 4 weeks after starting MMI treatment upon the switch to short days (Figure 4). Thyroid hormone levels remained low for the rest of the experiment. Body weight was unaffected by these low levels of thyroid hormones, indicating that MMI treatment had no major effects on the metabolic processes (Supplementary Figure 2). In the control group T4 and T3 significantly decreased during the photosensitive period compared to the photorefractory period. After switching back to long days, T4 concentrations significantly increased again to levels comparable to the photorefractory period, while T3 concentrations remained low.

**FIGURE 5:**
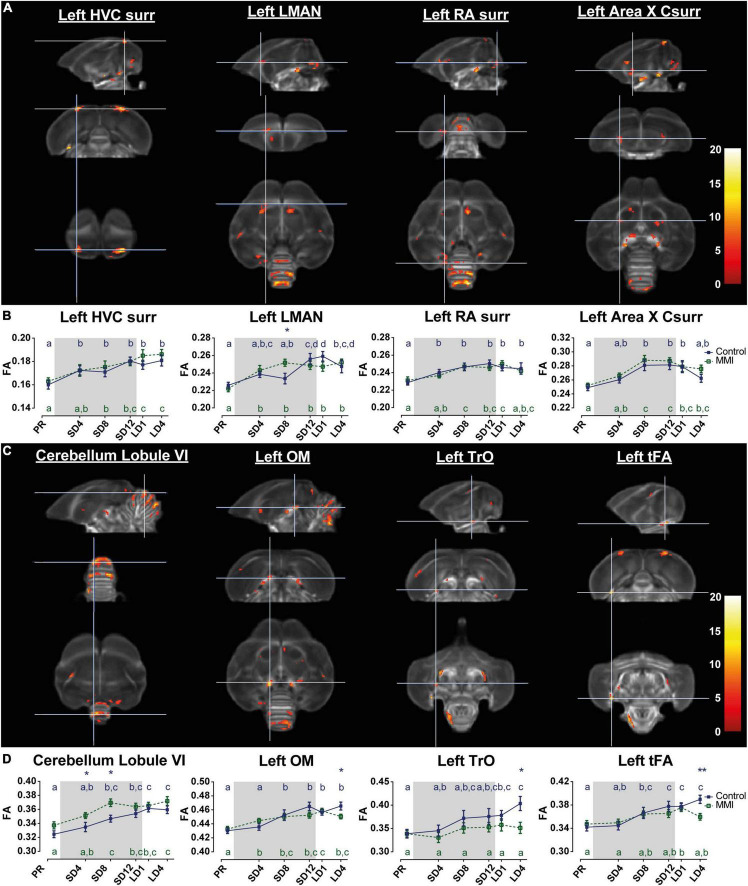
Voxel-based flexible factorial analysis revealed time effect in the song control system **(A)**, cerebellum and several white matter tracts **(C)**. The longitudinal changes over time of the extracted diffusion parameter fractional anisotropy (FA) of each ROI-based cluster is displayed below **(B,D)**. The statistical maps were assessed at p_uncorr_ < 0.001 and k_E_ ≥ 20 voxels with a small volume correction including regions of the song control system, white matter structures and the cerebellum. Significant voxels are color coded according to their F–values displayed on the scale on the right. The gray area indicates the photosensitive period of short days (8L:16D). *Post-hoc* statistical testing with Tukey’s HSD multiple comparison (*p* < 0.05) correction revealed significant differences between different time points, visualized by different letters. If two time points share the same letter, the DTI values are not significantly different from each other. Significant group differences at specific time points are indicated by *(p < 0.05) or ** (p < 0.01). Error bars shown are the standard error of the mean. Csurr, caudal surroundings; surr, surroundings.

In order to determine whether this thyroid hormone deprivation affected neuroplasticity, a ROI-based analysis identified the volumetric changes in song nuclei Area X and RA (Figures 4C,D). Additionally, a voxel-based factorial analysis was performed to establish the general FA changes over time and interaction between groups and time. The full results are summarized in Table 2 and the most prominent regions are summarized in Figure 5. From each significant cluster, we extracted the mean FA values and plotted them over time to determine the profile of temporal changes in FA (Figures 5B,D). Additionally, we extracted other diffusion parameters (mean, axial and radial diffusivity) and fixel-based measures from these ROIs to provide more insight into the basis of the FA changes (Figure 5 and Supplementary Figures 3–6).

**TABLE 2:** Overview interaction and time effect in FA in small volume correction.

Effect	Cluster	Hemisphere	Cluster		Peak	
			*p*_FWE_	k_E_	*p*_FWE_	*F*
Interaction time × group	OM	Left	0.066	25	0.017	8.71
Main time effect	HVC surroundings/HVC shelf	Left	<0.0001	80	<0.0001	14.28
		Right	<0.0001	233	<0.0001	16.71
	OM	Left	<0.0001	196	<0.0001	13.45
		Left	0.001	63	0.007	9.18
		Right	<0.0001	124	0.002	9.98
		Right	0.032	30	0.003	9.72
	Area X rostral surroundings	Left	0.007	41	0.266	7.12
		Right	<0.0001	123	<0.0001	10.86
	Area X caudal surroundings	Left	0.002	52	0.054	8.04
		Right	<0.0001	94	0.533	6.55
	tFA	Left	0.001	57	<0.0001	16.39
		Right	0.003	49	0.001	10.14
	Optic tract	Left	0.004	45	<0.0001	17.02
		Left	0.037	29	0.003	9.74
		Right	0.003	48	0.001	10.39
	RA surroundings/RA cup	Left	<0.0001	96	0.014	8.80
		Left	0.008	40	0.038	8.25
		Left	0.028	31	0.025	8.48
		Right	0.077	24	0.043	8.18
	HVC-RA tract	Left	0.003	49	0.053	8.06
		Right	0.002	52	0.021	8.59
	LMAN	Left	<0.0001	157	0.026	8.45
	Lamina mesopallialis	Left	0.003	49	0.024	8.49
		Left	0.001	57	0.132	7.54
		Right	0.057	26	0.039	8.23
	Caudal NCM	Left	0.011	38	0.225	7.24
	Cerebellum	Lobule VIII	<0.0001	528	<0.0001	20.20
		Lobule VII	<0.0001	741	<0.0001	19.92
		Lobule VIa	<0.0001	271	<0.0001	13.21
		Left side	<0.0001	255	<0.0001	13.15
		Lobule VI	<0.0001	419	<0.0001	12.46

*Table summarizing the significance at cluster level and peak level. P-values are FWE corrected. K_E_ indicates the number of voxels within a cluster. Gray values indicate clusters that are significant at cluster level but not at peak level.*

In general both groups presented similar neuroplasticity changes, both in volume and diffusion parameters, starting during the photosensitive period, as thyroid hormones decline. In line with the prior longitudinal MRI study (Orije et al., 2021a), the FA value of several parts of the song motor pathway, anterior forebrain pathway but also visual system and molecular layer of the cerebellum increase gradually over time. *Post-hoc* linear mixed model analysis revealed subtle interactions over time between the different groups. In the MMI group FA values in LMAN significantly increased at SD4, whereas FA values in the control group only increased at the end of the photosensitive period. Most interestingly, several tracts including the occipito-mesencephalic tract (OM), the fronto-arcopallial tract (tFA) and the optic tract (TrO) have similar *post-hoc* interactions, where the control group increased in FA after 4 weeks of photostimulation (LD4), but the MMI-treated group did not. This increase in FA was mainly linked to a decrease in radial diffusivity at LD4. At LD4 there was a significant difference between both groups in FA and radial diffusivity (Supplementary Figure 5). This indicates that the increase in FA at LD4 in the control group was most probably due to increased myelination of these tracts, something that is missing in the MMI-treated group.

**FIGURE 6:**
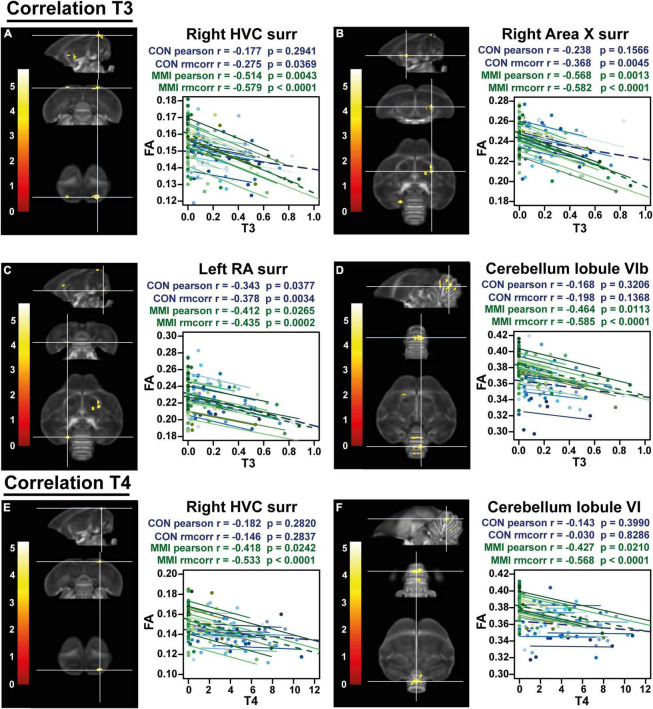
Overview of structural neural correlates of thyroid hormones T3 **(A–D)** and T4 **(E,F)** levels to fractional anisotropy (FA) in both groups identified using voxel-based multiple regression. The statistical maps were assessed at p_uncorr_ < 0.001 and k_E_ ≥ 20 voxels with a small volume correction including regions of the song control system, white matter structures and the cerebellum. Significant voxels are color coded according to their T–values displayed on the scale on the left. Next to each statistical parametric map, the identified correlations were further explored with repeated measures correlation of the control group (blue) and MMI treatment group (green). Solid colored lines show the best linear fit for the within-subject correlation in the control group (blue) and MMI group (green) using parallel regression lines with different shades for individual animals. The blue and green dashed line represent the linear fit of the overall Pearson correlation representing the between-subject correlations of the control and MMI group respectively. For bilateral correlations, only the left side is shown. surr, surroundings.

Remarkably, the inverse relationship between thyroid hormones and neuroplasticity is further confirmed by a voxel-based multiple regression, where the FA values in the surroundings of the song control nuclei and cerebellum present a negative correlation to the levels of circulating T3 and T4 (Figure 6 and Table 3). The extracted FA values were used to determine the within and between-subject correlation of each group separately. This way, we tried to distinguish whether the correlation was present in both groups. Right HVC, left RA, right Area X surroundings and cerebellar lobules VI-VII were negatively correlated to the circulating T3 levels in both the MMI and control group. As circulating T3 levels decreased during the photosensitive period, whether naturally in the control group or artificially in the MMI-treated group, FA values increased in these regions. This correlation was mostly driven by a between and within-subject correlation in the MMI-treated group (Table 3). Only the RA surroundings or RA cup had a significant negative between and within-subject correlation in the control group. Additionally, the control group had significant within-subject correlations to T3 at the level of the right HVC and caudal part of Area X surroundings. Correlations between T4 and the FA values at HVC surroundings or HVC shelf and cerebellum only existed in the MMI-treated group. Rather than the natural biological variation in T4 in the control group, it is the artificial lowering of the T4 levels by the MMI treatment that correlated to the neuroplasticity in these regions.

**TABLE 3:** Summary of the overall and repeated measures correlation of thyroid hormones T3 and T4 for control and MMI-treated group separately.

			Control group				MMI group			
	Cluster	Hemi-sphere	Pearson		rmcorr		Pearson		Rmcorr	
			*r*	*p*	*r*	*p*	*r*	*p*	*r*	*p*
T3	HVC surr/shelf	Right	–0.177	0.2941	**–0.275**	**0.0369**	**–0.514**	**0.0043**	**–0.579**	<**0.0001**
	Area X Csurr	Right	–0.238	0.1566	**–0.368**	**0.0045**	**–0.568**	**0.0013**	**–0.582**	**<0.0001**
	RA surr/cup	Left	**–0.343**	**0.0377**	**–0.378**	**0.0034**	**–0.412**	**0.0265**	**–0.435**	**0.0002**
	Cerebellum	Lobule VIb	–0.168	0.3206	–0.198	0.1368	**–0.464**	**0.0113**	**–0.585**	<**0.0001**
		Lobule VI	–0.037	0.8287	–0.026	0.8481	**–0.567**	**0.0013**	**–0.661**	**<0.0001**
		Lobule VIa	–0.150	0.3752	**–0.307**	**0.0192**	**–0.522**	**0.0037**	**–0.610**	<**0.0001**
		Lobule VII	–0.191	0.2564	–0.258	0.0501	**–0.378**	**0.0434**	**–0.509**	**<0.0001**
T4	HVC surr/shefl	Right	–0.182	0.2820	–0.146	0.2837	**–0.418**	**0.0242**	**–0.533**	<**0.0001**
	Cerebellum	Lobule V	–0.143	0.3990	–0.030	0.8286	**–0.427**	**0.021**	**–0.568**	**<0.0001**

*This table summarizes for each significant cluster-based ROI the overall Pearson’s correlation, the repeated measures correlation (rmcorr) and its significance for control and MMI-treated group separately. Voxel-based correlations in gray are significant at the cluster level, but not at peak level. Significant correlations are indicated in bold.*

In conclusion, the decline in circulating thyroid hormones during the photosensitive period, whether naturally or artificially, is negatively related to the microstructural neuroplasticity, specifically at the level of the song control system and cerebellum. On the other hand, high thyroid hormone levels during the photostimulated period appear to be necessary for the myelination and stabilization of several tracts.

### Hypothyroidism Prevents Reproductive Maturation and Affects Song Behavior

**FIGURE 7:**
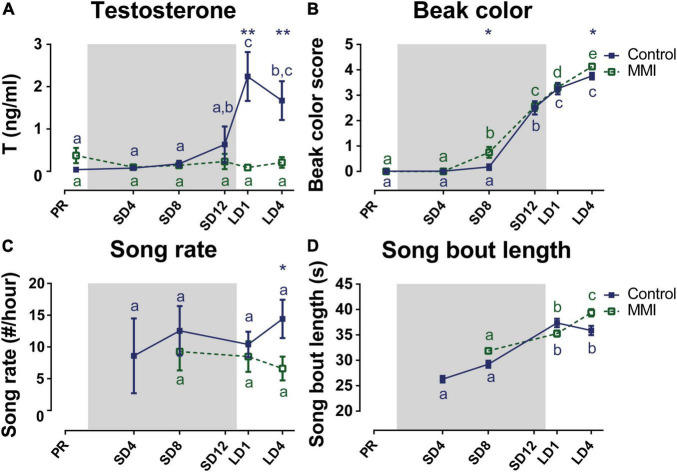
Overview of the seasonal changes in testosterone plasma levels **(A)**, beak color **(B)**, song rate **(C)**, and song bout length **(D)** in control and MMI-treated starlings. Solid and dashed lines represent the group average of control and MMI-treated starlings respectively with standard errors of the mean error bars. The gray area indicates the photosensitive period of short days (8L:16D). Different letters denote significant differences by comparison with each other in *post-hoc t*-tests with *p* < 0.05 (Tukey’s HSD correction for multiple comparisons) comparing the different time points to each other. If two time points share a common letter, the factors are not significantly different from each other.

Additionally, we looked at the effects of hypothyroidism on circulating testosterone levels and song behavior, since these are known to be related to seasonal neuroplasticity. Under natural conditions the HPG-axis matured upon photostimulation as shown by the elevated plasma testosterone levels, whereas in the hypothyroid group circulating testosterone levels remained low throughout the different photoperiods (Figure 7A). This indicates that thyroid hormones are required for the maturation of the reproductive axis. However, the beak color of MMI-treated birds turned yellow at the end of the photosensitive phase and in the photostimulated phase just like in the control group (Figure 7B). Beak color does not directly represent the amount of circulating testosterone, but rather indicates that testosterone is present in circulation, even when in low doses below the sensitivity limit of the RIA assay (Ball and Wingfield, 1987; Wingfield and Silverin, 2002).

Next, we looked at how this hormone modulation, especially the lack of high testosterone levels during photostimulation, affected the song behavior. The song rate did not change over time nor did it correlate to hormone levels in either group. Only after 4 weeks of photostimulation, MMI-treated starlings had a lower song rate compared to the untreated male starlings (Figure 7C). Interestingly, the song bout length of hypothyroid and control starlings evolved similarly over time, until the photostimulated period LD4. While under natural conditions the song bout length stabilized during the photostimulated period, the MMI-treated starlings further increased their song bout length from 35.26 ± 0.62 s at LD1 to 39.36 ± 0.71 s at LD4 (Figure 7D). Furthermore, song bout length was within-subjects positively correlated with the testosterone concentration in the control group (rmcorr = 0.572, *p* = 0.0054). Since testosterone plasma concentration in the MMI-treated group did not change over different seasons, it was not correlated to song bout length. However, beak color, which is a sensitive indicator of the presence or absence of circulating testosterone, did positively correlate to song bout length in both groups (control: rmcorr = 0.592, *p* = 0.0015; MMI: rmcorr = 0.4136, *p* = 0.0287).

**FIGURE 8:**
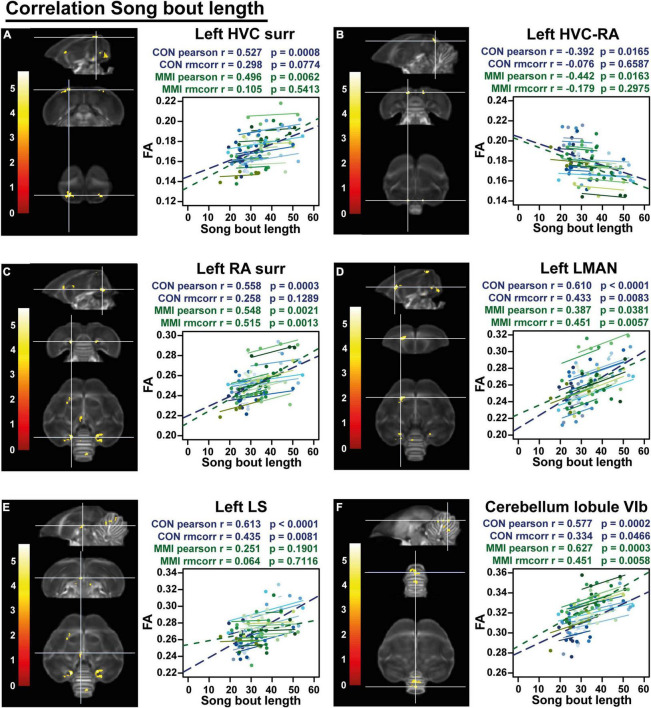
Overview of structural neural correlates of song bout length to fractional anisotropy (FA) in both groups identified using voxel-based multiple regression **(A–F)**. The statistical maps were assessed at p_uncorr_ < 0.001 and k_E_ ≥ 20 voxels with a small volume correction including regions of the song control system, white matter structures and the cerebellum. Significant voxels are color coded according to their T–values displayed on the scale on the left. Below each statistical parametric map, the identified correlations were further explored with repeated measures correlation of the control group (blue) and MMI treatment group (green). Solid colored lines show the best linear fit for the within-subject correlation in the control group (blue) and MMI group (green) using parallel regression lines with different shades for individual animals. The blue and green dashed line represent the linear fit of the overall Pearson correlation representing the between-subject correlations of the control and MMI group respectively. For bilateral correlations, only the left side is shown. surr, surroundings.

Finally, a voxel-based multiple regression analysis identified the neuronal correlates of the song bout length within the brain. A positive correlation to FA values was found at the level of the left HVC shelf, RA surroundings/cups (bilaterally), left LMAN, part of the left lateral septum (LS) and cerebellar lobules VI-VII whereas a cluster in the superior part of the HVC-RA tract near the HVC was negatively correlated to the song bout length (Figure 8 and Table 4). Both groups had significant between-subject correlations in all regions, except for the LS that only had a significant between-subject and within-subject correlation in the control group but not in the MMI-treated group (Figure 8 and Table 4). The neuronal correlate to song bout length at the level of the HVC was largely driven by a between-subject correlation, meaning that birds with longer song bout lengths, had higher FA values. On the other hand, FA values of the right RA, LMAN and cerebellar lobules VI-VII were correlated on a between- and within-subject level in both the control and MMI-treated group, indicating that the variation over time in song bout length was also correlated to the variation in structural neuroplasticity (measured by FA) on a subject level.

**TABLE 4:** Summary of the overall and repeated measures correlation of song bout length for control and MMI-treated group separately.

			Control group				MMI group			
	Cluster	Hemi-sphere	Pearson		rmcorr		Pearson		Rmcorr	
			*r*	*p*	*r*	*p*	*r*	*p*	*r*	*p*
Song bout length	RA surr/cup	Left	**0.558**	**0.0003**	0.258	0.1289	**0.548**	**0.0021**	**0.515**	**0.0013**
		Right	**0.575**	**0.0002**	**0.416**	**0.0117**	**0.481**	**0.0082**	**0.371**	**0.0258**
	HVC surr/shelf	Left	**0.527**	**0.0008**	0.298	0.0774	**0.496**	**0.0062**	0.105	0.5413
	LMAN	Left	**0.610**	<**0.0001**	**0.433**	**0.0083**	**0.387**	**0.0381**	**0.451**	**0.0057**
	Cerebellum	Lobule VIa	**0.366**	**0.0258**	**0.385**	**0.0206**	**0.596**	**0.0006**	**0.490**	**0.0024**
		Lobule VIb	**0.577**	**0.0002**	**0.334**	**0.0466**	**0.627**	**0.0003**	**0.451**	**0.0058**
		Lobule VII	**0.450**	**0.0053**	**0.370**	**0.0262**	**0.428**	**0.0206**	**0.442**	**0.0070**
		Lobule VIII	**0.360**	**0.0287**	**0.440**	**0.0073**	**0.566**	**0.0014**	**0.460**	**0.0047**
	LS	Left	**0.613**	<**0.0001**	**0.435**	**0.0081**	0.251	0.1901	0.064	0.7116
	HVC-RA-tract	Left	**–0.392**	**0.0165**	–0.076	0.6587	**–0.442**	**0.0163**	–0.179	0.2975
		Right	**–0.399**	**0.0145**	–0.153	0.4070	**–0.443**	**0.0162**	**–0.380**	**0.0221**

*This table summarizes for each significant cluster-based ROI the overall Pearson’s correlation, the repeated measures correlation (rmcorr) and its significance for control and MMI-treated group separately. Voxel-based correlations in gray are significant at the cluster level, but not at peak level. Significant correlations are indicated in bold.*

Overall, MMI treatment does not only decrease thyroid hormone but also prevent elevated testosterone plasma concentrations upon photostimulation, indicating that thyroid hormones indeed influence HPG-axis physiology in starlings as hypothesized previously based on work in other species (Yoshimura, 2013). This lack of high circulating testosterone levels could explain some of the effects on song behavior during photostimulation, like the lower song rate compared to the control group and the lack of song bout length stabilization. However, testosterone was not totally eliminated, as can happen with castration, as indicated by the beak color changing to yellow in the MMI treated birds. This change in beak color correlated to song bout length, which in turn had neuronal correlates at the level of several song control nuclei, the LS and cerebellum. This suggests that even low doses of testosterone, as reflected by beak color, can stimulate the song bout length and associated microstructural changes within the song control system and cerebellum.

## Discussion

**FIGURE 9:**
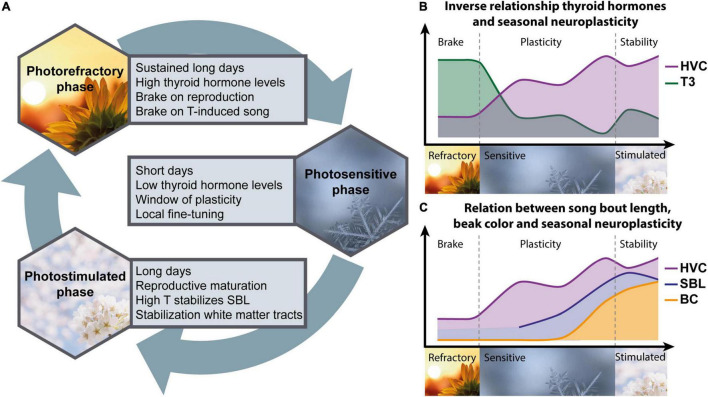
Schematic overview of the main interactions between thyroid hormones, song behavior and neuroplasticity across the different seasons **(A)**. During the photorefractory period, circulating thyroid hormone levels are high, sustaining brakes on reproductive maturation and testosterone (T) induced singing behavior. These brakes are lifted as the day length and thyroid hormones decrease during the photosensitive period, leading to a window of neuroplasticity, indicated by the inverse relationship between thyroid hormones and microstructure changes in song control nuclei like HVC **(B)**. Furthermore, during this photosensitive period beak color (BC), song bout length (SBL) and HVC microstructure start to increase gradually and are positively correlated to each other **(C)**. Even though the circulating concentrations of T and thyroid hormones are low, their effects might be maximized by local fine-tuning through upregulation of aromatase in the telencephalon (Riters et al., 2001) and thyroid hormone transporter LAT1 in HVC. Subsequent long day photostimulation will result in reproductive maturation, which requires thyroid hormones. The resulting peak in circulating testosterone stimulates the song rate and stabilizes the song bout length. High DIO3 expression in the HVC represses the potential activity of T3 or T4, preventing further neuroplasticity in order to promote song stability. However, in other parts of the brain high T4 is required for myelination and stabilization of several white matter tracts.

Firstly, this study established that there are indeed thyroid hormone regulating genes expressed in the adult songbird brain, including the song control system. Moreover, their seasonally changing expression pattern within the HVC suggests a direct mechanism in which thyroid hormones could modulate seasonal song behavior and neuroplasticity. High *LAT1* expression during the photosensitive phase points to an increased uptake of thyroid hormones in the HVC during this sensitive window of neuroplasticity, whereas during photostimulation, elevated *DIO3* expression limits the amount of active thyroid hormone, presumably to sustain song stability during the breeding season. Secondly, we further established the effects of thyroid hormones on song behavior and neuroplasticity, by inducing hypothyroidism (summarized in Figure 9). The artificially induced decline in thyroid hormones is more drastic than the natural decrease in thyroid hormones during the photosensitive period, but does not affect the onset of multisensory neuroplasticity, as both microstructural and volumetric changes still occur. In fact, the inverse relationship between thyroid hormones and neuroplasticity was further demonstrated by a negative correlation between plasma T3 and the microstructural changes at the level of several song control nuclei and cerebellum. However, persisting hypothyroidism did prevent the myelination and stabilization of several tracts during the photostimulated period. Furthermore, hypothyroidism also inhibited the photostimulation-induced increase in testosterone concentrations in the blood, indicating that as in Japanese quail thyroid hormones play a role in the activation of the HPG-axis upon photostimulation in starlings. The effect of thyroid hormones on testosterone is important for the interpretation of song behavioral changes, since testosterone is known for its effect on song motivation and song quality measures like song bout length (Alward et al., 2013). However, it is important to note that even low concentrations of testosterone appear sufficient to induce changes in song bout length and the associated seasonal neuroplasticity in songbirds (Tramontin et al., 2001; Caro et al., 2005). The presence of a yellow beak in the MMI treated birds meant that there was still some testosterone in the circulation (Ball and Wingfield, 1987), presumably enough to induce seasonal neuroplasticity.

We hypothesize that a global reduction of circulating thyroid hormones might be necessary to lift the brake on neuroplasticity imposed by the photorefractory period, whereas local fine-tuning of thyroid hormone and testosterone concentration through an upregulation of thyroid hormone transporters LAT1 and aromatase activity, respectively, could activate certain genes and mechanisms associated with neuroplasticity. However, the effects of thyroid hormones are complicated by their effect on the HPG-axis and the difference between circulating and intracellular thyroid hormone levels. Therefore, this study is just one of the first steps to disentangle the influence of thyroid hormones on seasonal neuroplasticity and provides a framework for future studies to further investigate the molecular changes induced by thyroid hormones in a seasonal songbird.

### Seasonal Local Fine-Tuning of Thyroid Hormone Regulating Genes in HVC

**FIGURE 10:**
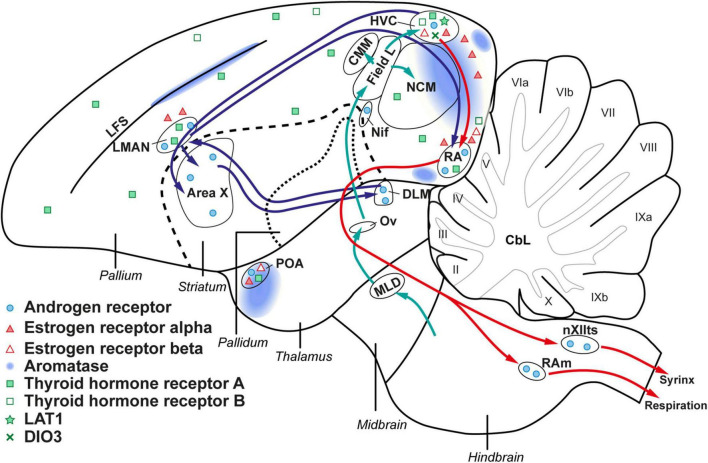
Schematic overview of the steroid and thyroid hormone receptors within the songbird brain. Androgen receptor, estrogen receptor, and aromatase expression is based on prior studies in starlings and canaries (Gahr, 1990; Bernard et al., 1999; Metzdorf et al., 1999). The expression of thyroid hormone regulating genes is derived from our own results.

Among the different thyroid hormone regulating genes expressed within the songbird brain (represented in a schematic overview in Figure 10), the seasonal changes in *DIO3* and *LAT1* at the level of the HVC support the hypothesis that thyroid hormones contribute to the seasonal neuroplasticity related to song behavior. During the photosensitive phase circulating thyroid hormones are low, but a local upregulation of thyroid hormone transporter *LAT1*, and downregulation of *DIO3* at the level of the HVC, suggests that the local thyroid hormone concentration in the HVC is higher during the photosensitive phase. The downregulation of *DIO3* is not matched by an upregulation of *DIO2*, since there is an apparent lack of *DIO2* expression in the entire telencephalon. It must be taken into account that ISH measures mRNA levels, which do not necessarily correlate to active protein levels due to further translational and post-translational regulation. All vertebrates need at least a minimum of brain *DIO2* activity to survive but research in chicken and zebra finches already indicated that *DIO2* expression in the brain decreases with age (Van Herck et al., 2015; Raymaekers et al., 2017). Our data seem to confirm that at least in adult birds, *DIO2* expression is too low to be detected by our ISH staining method. This is in stark contrast with what was found during developmental neuroplasticity in the song control nuclei in zebra finches, where the expression of *DIO2* remained high during the sensitive and sensorimotor learning phases (Raymaekers et al., 2017). Several other studies have further suggested that local presence of DIO2 is essential for critical period learning or that correct local balancing of active THs is essential in plastic brain areas involved with learning (Guadaño-Ferraz et al., 1999; Yamaguchi et al., 2012). In zebra finches DIO2 was most intensively expressed in endothelial cells of blood vessels. Potentially, angiogenesis and hormone supply *via* the blood-brain barrier are more important and pronounced during developmental neuroplasticity than for adult seasonal neuroplasticity.

Interestingly, several microarray studies in other seasonal songbirds have shown *DIO2* expression in HVC and RA with seasonal changes in expression. Stevenson et al. (2012) found that in starlings the *DIO2* expression in RA was the highest during the photosensitive phase, which is in line with our hypothesis of local upregulation of thyroid hormone regulating genes in the song control system during the non-breeding periods. There are however some species differences as Gambel’s white crowned sparrows have upregulated *DIO2* expression during breeding conditions in HVC and RA relative to short day conditions (Thompson et al., 2012).

The inverse ratio between thyroid hormone transporter *LAT1* and *DIO3* is seen during the photostimulated phase. Even though circulating T4 levels are elevated during this phase, the influx of thyroid hormones and the potential activity of T3 seems to be repressed in the HVC. Local DIO2 and DIO3 activity can fine-tune intracellular thyroid hormone concentration at least partially independent of the circulating thyroid hormone levels. For example in Japanese quail, reciprocal switching of *DIO2* and *DIO3* expression upon photostimulation can locally increase T3 concentration by a 10-fold, even though plasma concentrations do not change (Yoshimura et al., 2003). Interestingly, this could mean that, whereas increased uptake of thyroid hormones by *LAT1* induces neuroplasticity, *DIO3* activity is a possible factor for stability of neural tissue as would be expected in the photostimulated stage.

Apart from DIO3 and LAT1, our study also looked at the expression of thyroid hormone receptors and other thyroid hormone transporters. Surprisingly, other thyroid hormone transporters like *MCT8*, *MCT10*, or *OATP1C1*, were not detected by ISH in any photoperiod in the starling’s telencephalon, even though MCT8 is the transporter with the highest affinity for thyroid hormones (Bourgeois et al., 2016). Several studies, in mammals as well as birds, have shown that MCT8 is highly expressed in the developing brain and is considered as the most important thyroid hormone transporter during developmental neuroplasticity (Van Herck et al., 2013; Raymaekers et al., 2017). However, our data suggest that the situation might be different in adult birds, where *LAT1* seems to be more prominent as a mediator in seasonal neuroplasticity.

We found *THRA* expression in large parts of the pallium including HVC, RA and LMAN, whereas the striatum including Area X lacked *THRA* expression, which is in line with the expression pattern of *THRA* in adult zebra finches (Raymaekers et al., 2017). In contrast, *THRB* was not detected in any song control nucleus. These observations align with what has been observed in chickens: *THRA* expression seems to be widespread while *THRB* is more locally expressed and exerts some specific functions for brain development (Forrest et al., 1991; Bradley et al., 1992; Darras et al., 2011; Van Herck et al., 2013). Interestingly the expression pattern of *THRA* and *THRB* does not change seasonally, indicating that while at least *THRA* is necessary for proper genomic thyroid hormone action in the song control system, the level of thyroid hormone receptor expression is not a regulatory factor of song control system plasticity.

Together these findings suggest an active role for thyroid hormones during the non-breeding phase to induce plasticity in the song control system, in contrast to the photostimulated phase where thyroid hormones are locally decreased in the HVC to promote stabilization of the song.

### Complex Interaction Between Thyroid Hormones and Neuroplasticity

In the second experiment, we deprived male starlings of circulating thyroid hormones by MMI treatment during the photosensitive and photostimulated phase. MMI is an anti-thyroid drug inhibiting the synthesis of T3 and T4 at the level of the thyroid gland, which gradually depletes the circulating thyroid hormone levels. MMI treatment was started on the first short day of the photosensitive period, as earlier treatment would terminate the photorefractory period (Dawson et al., 1985). However, this also implies that during the first weeks of the photosensitive period there are still some circulating thyroid hormones present. After 4 weeks of MMI treatment thyroid hormone levels were successfully depleted, and remain minimal during the photostimulated phase. This is in line with findings in Japanese quail, where MMI treatment for 4 weeks resulted in decreased thyroid hormone levels, testes weight and testosterone levels (Weng et al., 2007). In immature rats MMI affected the HPG-axis, decreasing luteinizing hormone (LH) and testosterone plasma concentration, but also decreased the testes binding sites for LH by 30% (Valle et al., 1985; Pérez et al., 2018).

Deprivation of thyroid hormones during the photosensitive period did not prevent microstructural or volumetric neuroplasticity from occurring. In contrast, within the MMI group LMAN showed earlier significant differences in FA during the short days compared to the control group, pointing to an earlier onset of neuroplasticity coinciding with the earlier depletion of T3 and T4. This finding is further supported by the presence of THRA within LMAN, making it possible to respond directly to changes in thyroid hormone levels. The decrease of circulating thyroid hormones is necessary to terminate the photorefractory period for the reproductive system (Dawson et al., 1986). The photorefractory period depends on thyroid hormones to prevent reproductive maturation in response to long day length (Nicholls et al., 1988). The photoperiodic changes in song control nuclei are not exclusive to male starlings, also female starlings experience seasonal changes in song behavior, which have been associated to seasonal changes in song control nuclei (Van Meir et al., 2006; Orije et al., 2021b). Photorefractoriness not only forms a brake on gonadal maturation, but also prevents an increase in singing activity in female starlings in response to testosterone treatment, in contrast to photosensitive females treated with testosterone (Rouse et al., 2015). Potentially, the global reduction of circulating thyroid hormones is necessary to lift the brake on singing and on reproduction imposed by the photorefractory period.

Interestingly, circulating thyroid hormones present a negative within-subject correlation to neuroplasticity (indicated by FA changes) in HVC, Area X and RA surroundings both in the control and hypothyroid group, indicating that decreasing circulating thyroid hormone levels are associated with increased neuroplasticity during the photosensitive phase. This negative correlation between T3 and neuroplasticity seems in contrast to the thyroid hormone regulating genes in HVC, which supports an active role of thyroid hormones during the photosensitive phase. We hypothesize that while a global reduction in thyroid hormones is necessary to lift the brake imposed by the photorefractory period, local fine-tuning of thyroid hormone concentration through thyroid hormone regulating proteins like LAT1 and DIO3 could potentially activate mechanisms associated with neuroplasticity. A similar local fine-tuning of testosterone levels during the photosensitive phase has been suggested by Riters et al. (2001). They found increased aromatase activity and decreased 5β-reductase activity during the photosensitive period in male starlings, increasing the brain’s sensitivity to testosterone despite the low circulating testosterone levels.

At the end of the photosensitive phase, the sensitive window closes by imposing molecular brakes like perineuronal nets (PNNs) and myelin. PNNs are highly expressed in the HVC of adult zebra finches after their song learning and predicts song maturity (Balmer et al., 2009). However, starlings have far less PNNs, compared to closed-ended learning zebra finches, suggesting that PNN might play less of a role in the control of seasonal neuroplasticity of open-ended learning starlings (Cornez et al., 2017). Our prior studies suggested myelination rather than PNNs as another candidate mechanism that contributes to ending a sensitive window in open-ended learners like starlings (Orije et al., 2021a). In the control group, several tracts including OM, TrO and tFA increased in FA value upon photostimulation, due to decreased radial diffusivity, which implies an increase in myelination (Song et al., 2002, 2005; De Groof et al., 2008). This increased FA value was absent in the MMI-treated group, which could reflect the thyroid hormone effect on myelination. Myelin basic protein is a positively regulated thyroid hormone dependent gene, which could respond to the normally increased T4 plasma concentrations during the photostimulated phase. Furthermore, thyroid hormone therapy was shown to induce myelinogenesis in an animal model of demyelination (Harsan et al., 2008). So increased levels of testosterone and thyroid hormone levels during photostimulation could play a role in ending a sensitive window for plasticity in song behavior and its underlying neural structure through myelination acting as a molecular brake.

### Hypothyroidism Indirectly Affects the Song Behavior by Preventing Reproductive Maturation

Thyroid hormones not only have an influence during the photosensitive period, but also play a prominent role throughout the photostimulated phase. The present results show that hypothyroidism inhibited the photostimulation-induced increase in testosterone in starlings. Yoshimura et al. (2003) demonstrated that in Japanese quail thyroid hormones play a crucial role in the activation of the HPG-axis upon photostimulation. Exposure to long days increases hypothalamic T3 concentration as a result of a local upregulation of *DIO2* and downregulation of *DIO3* in the mediobasal hypothalamus. This locally produced bioactive T3 subsequently results in morphological changes in GnRH nerve terminals enabling GnRH secretion resulting in activation of the HPG-axis (Yoshimura, 2013). In starlings under natural photoconditions, this phenomenon was observed to a lesser extent, but it did not corroborate the proposed photoperiodic regulation of the HPG-axis through *DIO2/DIO3* expression (Bentley et al., 2013). It is suggested that both phylogenetic differences and the acuteness at which photoperiods are changed in experiments might influence the changes in hypothalamic thyroid hormone regulation. Likely, the regulatory mechanisms in natural conditions are similar but more gradual. In agreement with manipulation studies in quail where the DIO2 inhibitor iopanoic acid prevents testicular growth (Yoshimura et al., 2003), our manipulation study confirms the active role of thyroid hormones in the activation of the HPG-axis.

The absence of high testosterone levels in hypothyroid starlings during the photostimulated phase could explain some of the differences in song behavior, like the lower song rate and the lack of song bout length stability during the photostimulated period. Testosterone is known to increase the motivation to sing, by interacting with the medial preoptic nucleus (POM), since local testosterone implantation in the POM of castrated male canaries increased their song rate (Alward et al., 2013). Furthermore, testosterone is necessary for song crystallization and stabilization in sparrows, zebra finches and canaries (Marler et al., 1988; Korsia and Bottjer, 1991; Williams et al., 2003). Blocking testosterone by castration or by the androgen receptor antagonist flutamide, causes variability in song of zebra finches and canaries (Wang et al., 2014; Alward et al., 2017). More specifically blocking androgen receptors in the RA of male canaries, caused song duration to increase, whereas specific blocking in HVC increased the variability in used syllables (Alward et al., 2017).

The correlation between song bout length and testosterone is only present in the control group, since MMI treatment inhibited the photostimulation-induced increase in testosterone. However, beak color was still correlated to the song bout length in the MMI group, indicating that even low concentrations of testosterone, below detection limit of the RIA assay but enough to induce beak color changes, are linked to changes in song bout length. The neural substrate of song bout length includes song control nuclei in control of the quality of song performance like HVC and RA, which concurs with earlier correlation studies in starlings and other songbirds (Bernard et al., 1996; Garamszegi and Eens, 2004). Remarkably, also LMAN, a song nucleus that is not necessary for song production in adult birds, but is required for song learning and introducing song variability in juvenile zebra finches (Bottjer et al., 1984; Olveczky et al., 2005; Thompson and Johnson, 2007), correlated to song bout length changes within and between subjects. This corroborates that the seasonal increase in LMAN volume (reported by Stevenson and Ball, 2010) and microstructure is related to the increased song performance as a result of the yearly song remodeling, as proposed by Stevenson and Ball (2010). Moreover, each time a songbird learns a new song or adds new song phrases to its repertoire, the auditory responses in LMAN neurons are lost or overwritten (Yazaki-Sugiyama and Mooney, 2004). Furthermore, also other structures like the lateral septum and cerebellar lobule VI-VII are correlated to song bout length. At the level of the lateral septum Merullo et al. (2015) showed a positive correlation between neurotensin (a neuropeptide that regulates dopamine activity) expression and both sexually motivated song and non-vocal courtship behaviors in starlings. Whereas in songbirds, only few studies looked at the role of the cerebellum in song behavior through the cerebello-thalamic-basal ganglia pathway (Person et al., 2008; Nicholson et al., 2018; Pidoux et al., 2018), in humans, the cerebellum is known to be involved in language tasks (lobule VI and crus I/II) and phonological storage during the maintenance of verbal information (VIIb/VIII). A prior lesion study showed that lesioning Area X caused structural remodeling of the cerebello-thalamic-basal ganglia pathway extending into cerebellar lobules VIb and VII (Hamaide et al., 2020). Our results further corroborate that cerebellar lobules VI-VII retain the neural substrate for song behavior. Furthermore, these parts of the cerebellum show seasonal neuroplasticity, both in male and female starlings (Orije et al., 2021a).

Many early studies concluded that relatively high testosterone concentrations seem to be involved in high song rate and song stability during the photostimulated phase or the annual cycle. More recent studies have found that low concentrations of testosterone are sufficient to induce seasonal neuroplasticity (Tramontin et al., 2001; Caro et al., 2005). In our study, changes in beak color represented low concentrations of testosterone that are biologically active and correlated to song bout length, which has its neural substrate in several song control nuclei, lateral septum and cerebellum.

### Future Perspectives

MMI treatment did not only drastically reduce thyroid hormones to a minimum, it also inhibited the increase of testosterone upon photostimulation. In the current study we altered both thyroid and testosterone concentrations. Future studies might want to control the amount of circulating testosterone by castration or testosterone implantation during photostimulation, to be able to solely investigate the effects of thyroid hormone manipulation. This way the suggested effect of thyroid hormone on myelination of several tracts during the photostimulated phase could be attributed to the lack of thyroid hormone’s positive effect on myelination, if myelination is still affected with MMI treatment combined with testosterone supplementation during photostimulation.

Importantly, MMI treatment does not completely abolish thyroidal hormone production and takes a few weeks to fully deplete circulating thyroid hormone levels. Thus low concentrations of thyroid hormones, especially during the first weeks of the photosensitive period, could still be locally picked up within the brain, as suggested by the increased LAT1 expression in the HVC. Future studies could consider other manipulations to investigate the effects of thyroid hormones on neuroplasticity in songbirds and their song behavior. For example, iopanoic acid both inhibits thyroid hormone release from the thyroid gland but also the conversion of T4 to T3 by inhibiting DIO2, however it is less practical for long term treatment strategies (Pant and Chandola-Saklani, 1993). In the past both surgical and radioactive thyroidectomy have been applied to study the effects of thyroid hormones on seasonal reproduction (Dawson et al., 1986; Bentley et al., 2000). Analogous to experiments examining the local effects of testosterone by local androgen receptor inhibition (Alward et al., 2017), thyroid hormone antagonists and thyroid hormone receptor modulators have been developed and could be injected locally within specific song nuclei, to investigate their role in song behavior (Lim et al., 2002; Yoshihara and Scanlan, 2003; Raparti et al., 2013).

Future studies might also want to investigate whether thyroid hormones directly partake in the regulation of molecular mechanisms of reopening a sensitive window in vocal learning. Thyroid hormone function is complex, some genes are upregulated in the presence of T3 (positively regulated genes), whereas some genes are down-regulated (negatively regulated genes). Gil-Ibanez et al. (2017) showed that both positively and negatively regulated genes can play a role in neuroplasticity. Further studies are required to identify the genetic expression changes upon thyroid hormone manipulation in starling brain, and to establish how these genetic changes affect neuroplasticity.

## Data Availability Statement

The datasets presented in this study can be found in online repositories. The names of the repository/repositories and accession number(s) can be found below: Dryad (https://doi.org/10.5061/dryad.rjdfn2zd1).

## Ethics Statement

The animal study was reviewed and approved by the animal welfare regulations of the John Hopkins University of Baltimore, MD, United States and the Committee on Animal Care and Use of the University of Antwerp, Belgium (2014-52).

## Author Contributions

JO performed the MRI measurements and processing. SR performed the ISH experiments and processing. VD and AV were involved in the planning and supervision of the experiments. GM, GD, EJ, and MV supervised the practical work. GB provided the starling material for the ISH experiments. JO drafted the manuscript and designed the figures. All authors discussed the results and commented on the manuscript.

## Conflict of Interest

The authors declare that the research was conducted in the absence of any commercial or financial relationships that could be construed as a potential conflict of interest.

## Publisher’s Note

All claims expressed in this article are solely those of the authors and do not necessarily represent those of their affiliated organizations, or those of the publisher, the editors and the reviewers. Any product that may be evaluated in this article, or claim that may be made by its manufacturer, is not guaranteed or endorsed by the publisher.

## Footnotes

1
https://www.fil.ion.ucl.ac.uk/spm
