# Supplementary material for: Unraveling the Role of Thyroid Hormones in Seasonal Neuroplasticity in European Starlings (Sturnus vulgaris)
Source: Front Mol Neurosci. 2022 Jun 28;15:897039. doi: 10.3389/fnmol.2022.897039 (PMC9275473; doi:10.3389/fnmol.2022.897039)
Supplement: Supplementary file 1 [file Data_Sheet_1.pdf]

Supplementary Material

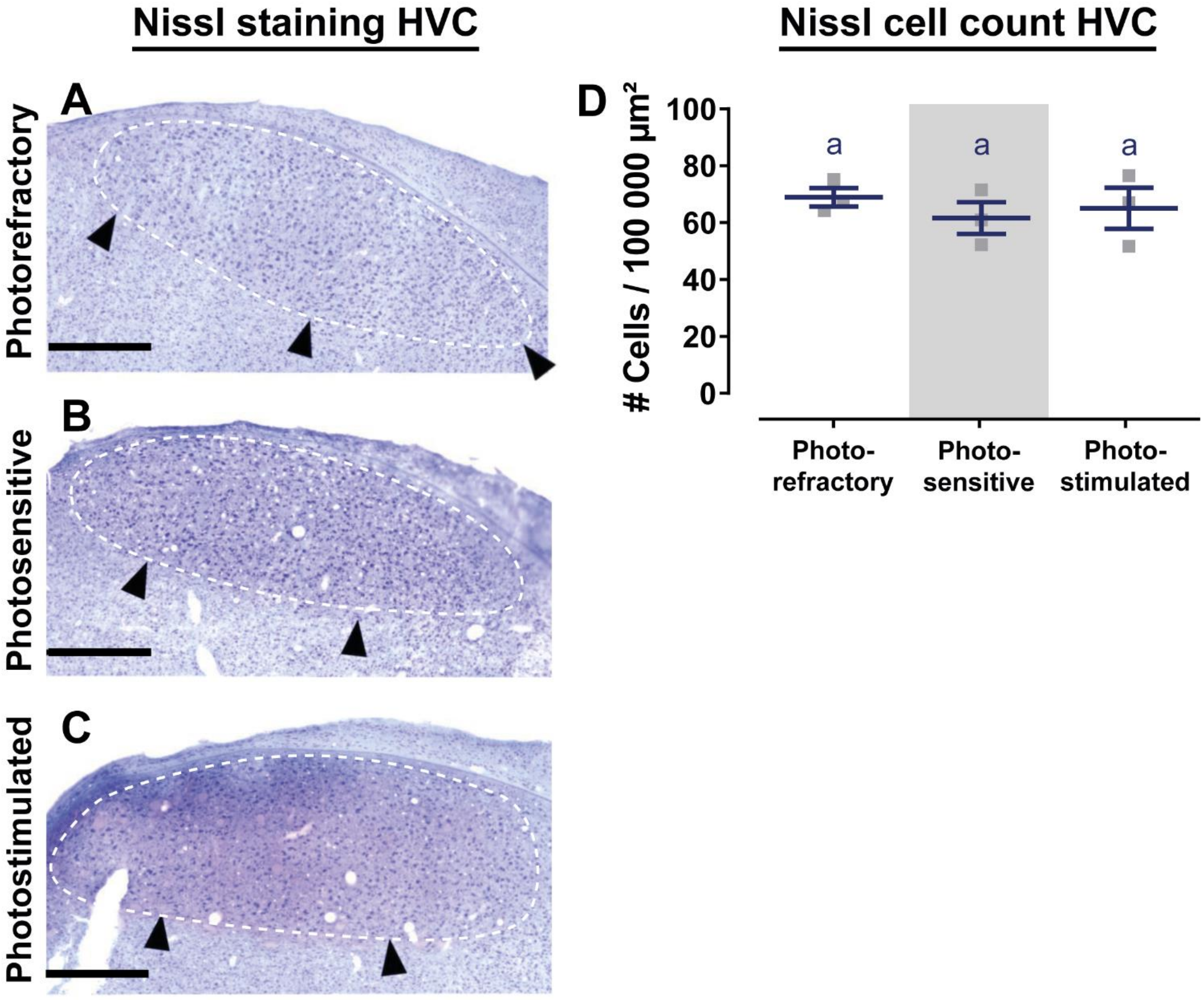

Supplementary Figure 1. Representative images of the HVC of starlings in different photoperiodic states stained by cresyl violet (A, B, C) and quantitative analysis of the Nissl cell count in the HVC. The border of HVC is designated by white dashed lines and black arrowheads. Scale bar = 500 µm. Horizontal bars represent the average with standard deviation error bars (n=3). The grey area indicates the photosensitive phase.

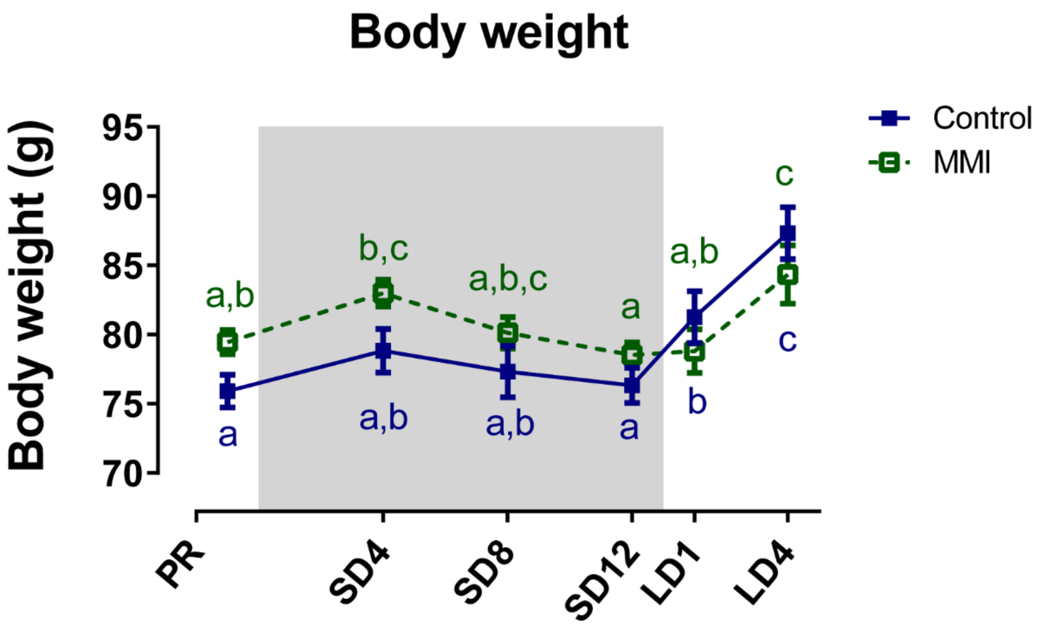

Supplementary Figure 2. Overview of the seasonal changes in body weight in control and MMI-treated starlings. Solid and dashed lines represent the group average of control and MMI-treated starlings respectively with standard errors of the mean error bars. The grey area indicates the photosensitive period of short days (8L:16D). Different letters denote significant differences by comparison with each other in post-hoc t-tests with $p < 0.05$ (Tukey's HSD correction for multiple comparisons) comparing the different time points to each other. If two time points share a common letter, the body weight is not significantly different.

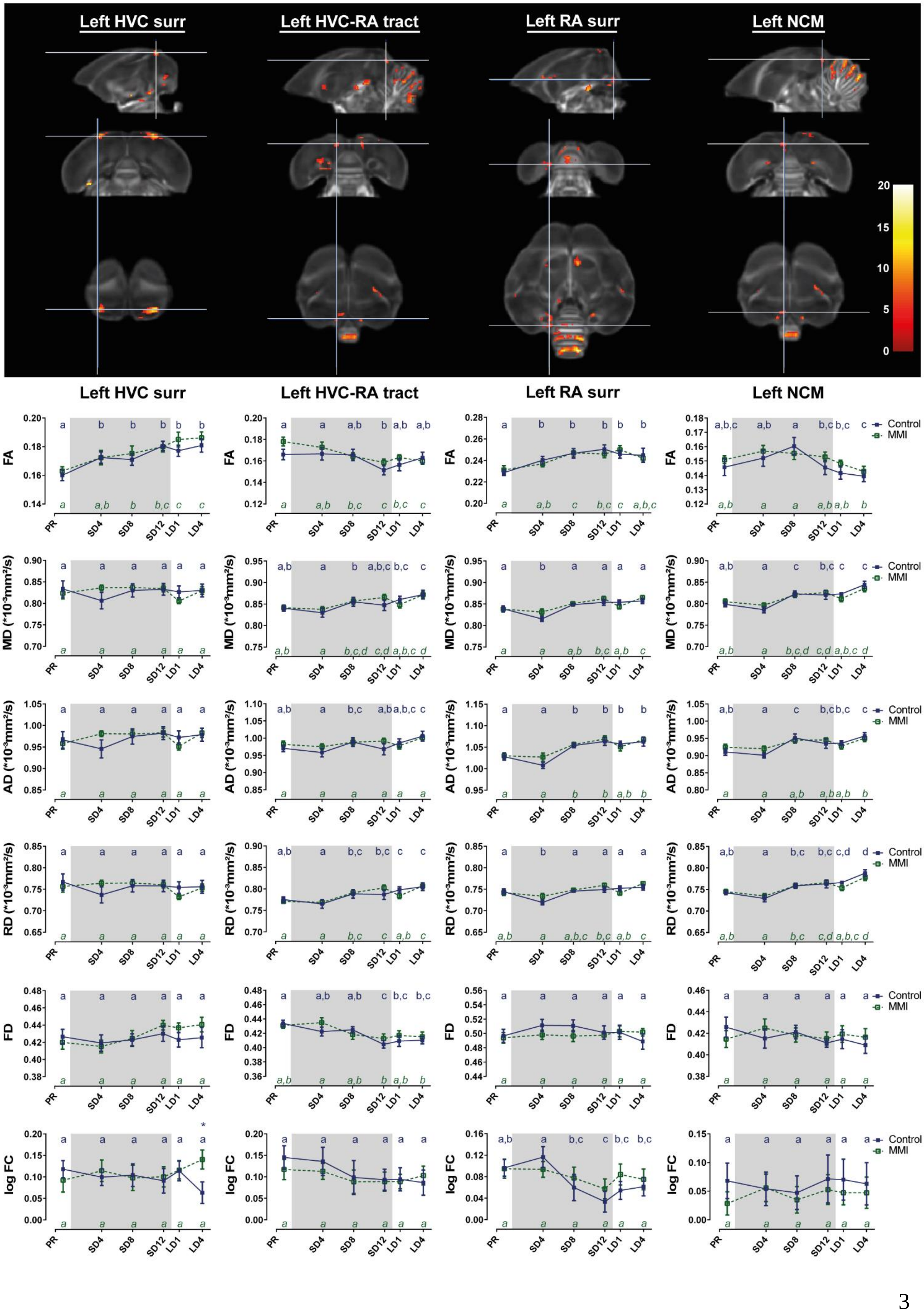

Left HVC surr
Left HVC-RA tract
Left RA surr
Left NCM
FA
MD (*10⁻³mm²/s)
AD (*10⁻³mm²/s)
RD (*10⁻³mm²/s)
FD
log FC
PR
SD4
SD8
SD12
LD1
LD4
Control
MMI

Supplementary Figure 3. Summary of the significant longitudinal changes over time in fractional anisotropy (FA), mean diffusivity (MD), axial diffusivity (AD), radial diffusivity (RD), fiber density (FD) and logarithmic transformed fiber-bundle cross-section (log FC) extracted from ROI-based clusters at level of the left HVC, HVC-RA tract, RA and NCM. The statistical maps were assessed at $p_{uncorr}<0.001$ and $k_E \geq 20$ voxels with a small volume correction including regions of the song control system, white matter structures and the cerebellum. Significant voxels are color coded according to their F–values displayed on the scale on the right. For bilateral time changes, only the left side is shown. The grey area indicates the photosensitive period of short days (8L:16D). Post hoc statistical testing with Tukey's HSD multiple comparison ($p < 0.05$) correction revealed significant differences between different time points, visualized by different letters. If two time points share the same letter, the DTI values are not significantly different from each other. Significant group differences at specific time points are indicated by *($p < 0.05$), ** ($p < 0.01$) or *** ($p < 0.001$). Error bars shown are the standard error of the mean. Abbreviations: surr, surroundings.

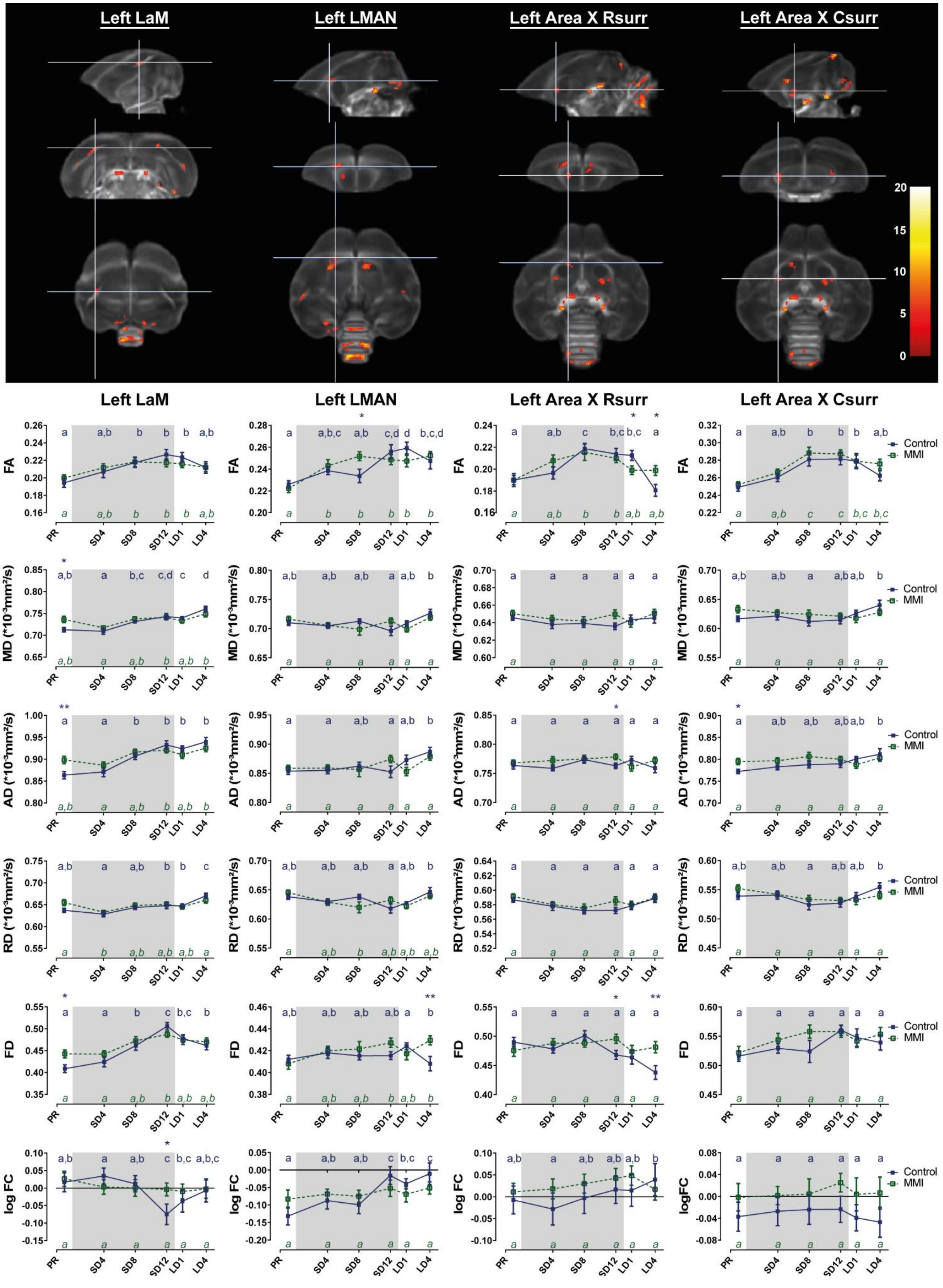

Left LaM
Left LMAN
Left Area X Rsurr
Left Area X Csurr
20
15
10
5
0
Left LaM
Left LMAN
Left Area X Rsurr
Left Area X Csurr
FA
MD (*10⁻³mm²/s)
AD (*10⁻³mm²/s)
RD (*10⁻³mm²/s)
FD
log FC
logFC
PR
SD4
SD8
SD12
LD1
LD4
Control
MMI

Supplementary Figure 4. Summary of the significant longitudinal changes over time in fractional anisotropy (FA), mean diffusivity (MD), axial diffusivity (AD), radial diffusivity (RD), fiber density (FD) and logarithmic transformed fiber-bundle cross-section (log FC) extracted from ROI-based clusters at level of the left LaM, LMAN, Area X rostral and caudal surroundings. The statistical maps were assessed at $p_{uncorr}<0.001$ and $k_E \geq 20$ voxels with a small volume correction including regions of the song control system, white matter structures and the cerebellum. Significant voxels are color coded according to their F–values displayed on the scale on the right. For bilateral time changes, only the left side is shown. The grey area indicates the photosensitive period of short days (8L:16D). Post hoc statistical testing with Tukey's HSD multiple comparison ($p < 0.05$) correction revealed significant differences between different time points, visualized by different letters. If two time points share the same letter, the DTI values are not significantly different from each other. Significant group differences at specific time points are indicated by *($p < 0.05$), ** ($p < 0.01$) or *** ($p < 0.001$). Error bars shown are the standard error of the mean. Abbreviations: Csurr, caudal surroundings; Rsurr, rostral surroundings.

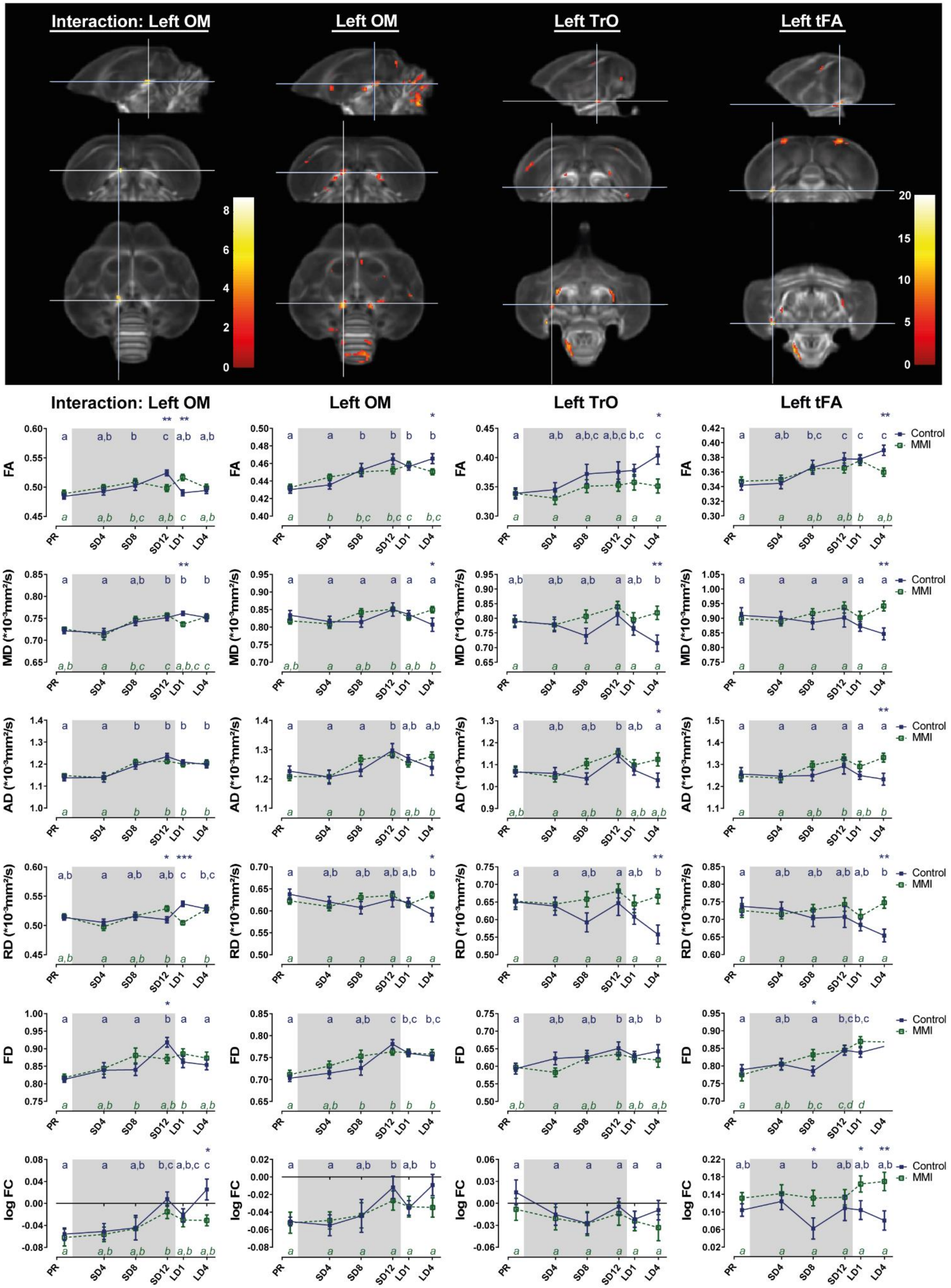
Interaction: Left OM
Left OM
Left TrO
Left tFA
FA
MD (*10⁻³mm²/s)
AD (*10⁻³mm²/s)
RD (*10⁻³mm²/s)
FD
log FC
PR
SD4
SD8
SD12
LD1
LD4
Control
MMI

Supplementary Figure 5. Summary of the significant longitudinal changes over time in fractional anisotropy (FA), mean diffusivity (MD), axial diffusivity (AD), radial diffusivity (RD), fiber density (FD) and logarithmic transformed fiber-bundle cross-section (log FC) extracted from ROI-based clusters at level of the left OM, TrO and tFA. The statistical maps were assessed at $p_{uncorr}<0.001$ and $k_E \geq 20$ voxels with a small volume correction including regions of the song control system, white matter structures and the cerebellum. Significant voxels are color coded according to their F–values displayed on the scale on the right. For bilateral time changes, only the left side is shown. The grey area indicates the photosensitive period of short days (8L:16D). Post hoc statistical testing with Tukey's HSD multiple comparison ($p < 0.05$) correction revealed significant differences between different time points, visualized by different letters. If two time points share the same letter, the DTI values are not significantly different from each other. Significant group differences at specific time points are indicated by *($p < 0.05$), ** ($p < 0.01$) or *** ($p < 0.001$). Error bars shown are the standard error of the mean.

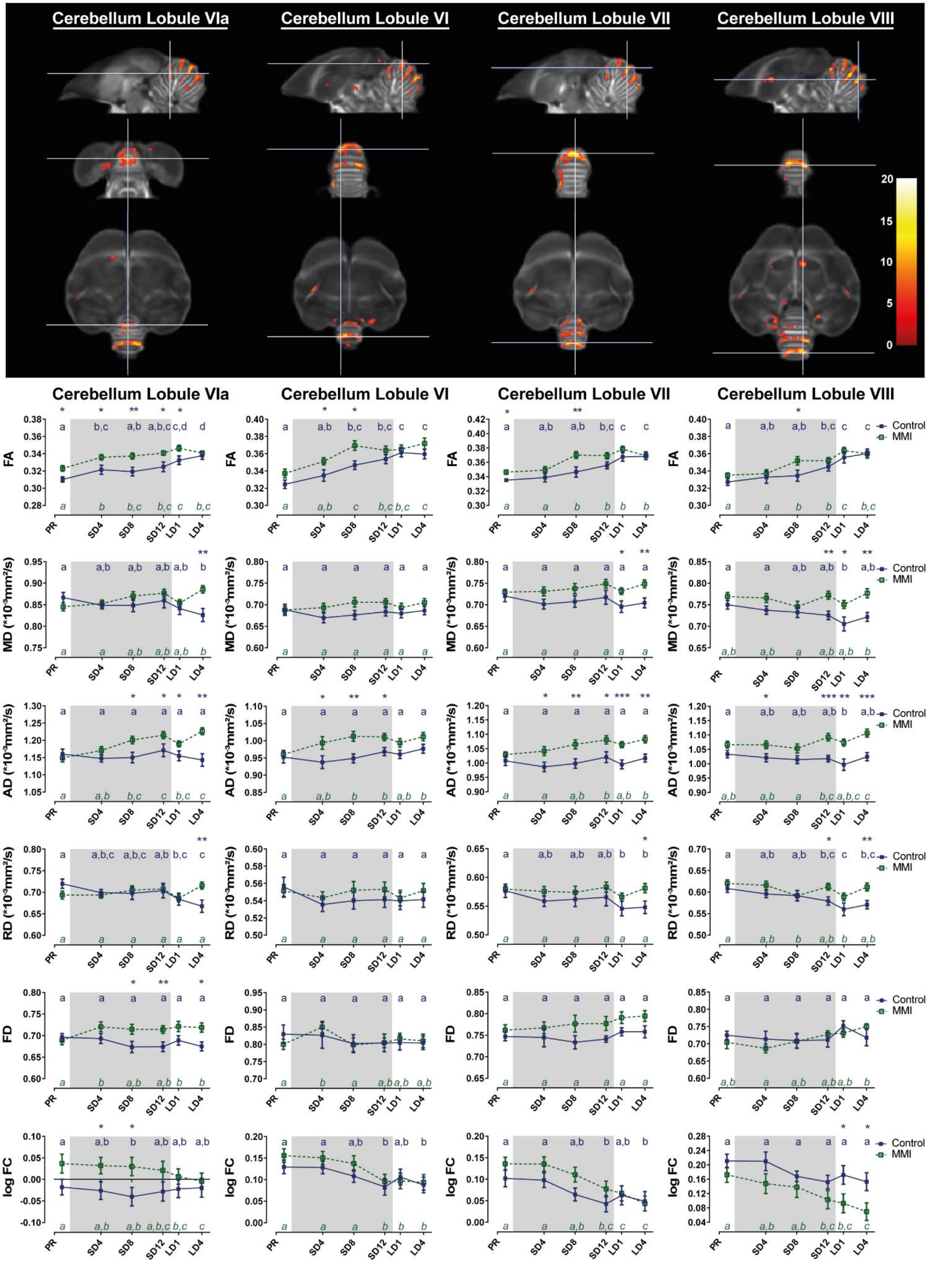
Cerebellum Lobule VIa
Cerebellum Lobule VI
Cerebellum Lobule VII
Cerebellum Lobule VIII
Control
MMI
FA
MD (*10⁻³mm²/s)
AD (*10⁻³mm²/s)
RD (*10⁻³mm²/s)
FD
log FC
PR
SD4
SD8
SD12
LD1
LD4

Supplementary Figure 6. Summary of the significant longitudinal changes over time in fractional anisotropy (FA), mean diffusivity (MD), axial diffusivity (AD), radial diffusivity (RD), fiber density (FD) and logarithmic transformed fiber-bundle cross-section (log FC) extracted from ROI-based clusters at level of the cerebellar lobules VIa, VI, VII and VIII. The statistical maps were assessed at $p_{uncorr}<0.001$ and $k_E \geq 20$ voxels with a small volume correction including regions of the song control system, white matter structures and the cerebellum. Significant voxels are color coded according to their F–values displayed on the scale on the right. For bilateral time changes, only the left side is shown. The grey area indicates the photosensitive period of short days (8L:16D). Post hoc statistical testing with Tukey's HSD multiple comparison ($p < 0.05$) correction revealed significant differences between different time points, visualized by different letters. If two time points share the same letter, the DTI values are not significantly different from each other. Significant group differences at specific time points are indicated by *($p < 0.05$), ** ($p < 0.01$) or *** ($p < 0.001$). Error bars shown are the standard error of the mean.
